# Construction of a synthetic metabolic pathway for biosynthesis of 2,4-dihydroxybutyric acid from ethylene glycol

**DOI:** 10.1038/s41467-023-37558-x

**Published:** 2023-04-06

**Authors:** Cláudio J. R. Frazão, Nils Wagner, Kenny Rabe, Thomas Walther

**Affiliations:** Institute of Natural Materials Technology, TU Dresden, 01062 Dresden, Germany

**Keywords:** Metabolic engineering, Environmental biotechnology, Applied microbiology, Metabolic engineering

## Abstract

Ethylene glycol is an attractive two-carbon alcohol substrate for biochemical product synthesis as it can be derived from CO_2_ or syngas at no sacrifice to human food stocks. Here, we disclose a five-step synthetic metabolic pathway enabling the carbon-conserving biosynthesis of the versatile platform molecule 2,4-dihydroxybutyric acid (DHB) from this compound. The linear pathway chains ethylene glycol dehydrogenase, D-threose aldolase, D-threose dehydrogenase, D-threono-1,4-lactonase, D-threonate dehydratase and 2-oxo-4-hydroxybutyrate reductase enzyme activities in succession. We screen candidate enzymes with D-threose dehydrogenase and D-threonate dehydratase activities on cognate substrates with conserved carbon-centre stereochemistry. Lastly, we show the functionality of the pathway by its expression in an *Escherichia coli* strain and production of 1 g L^−1^ and 0.8 g L^−1^ DHB from, respectively, glycolaldehyde or ethylene glycol.

## Abstract (web summary)

Ethylene glycol is an attractive two-carbon alcohol substrate for bioproduction as it can be derived from CO2 or syngas. Here, the authors design a five-step synthetic metabolic pathway in *E. coli* to enable the carbon-conserving biosynthesis of the platform chemical 2,4-dihydroxybutyric acid from ethylene glycol.

## Introduction

The reduction of our dependence on fossil resources achieved through the sustainable manufacture of bio-fuels and chemicals is the cornerstone of a bioeconomy^1^. However, many industrial bioprocesses are based on energy plant-derived sugars^2,3^, and thus compete directly with available human food stocks. In this context, the use of ethylene glycol (EG) receives growing interest. This inexpensive C_2_ compound can be obtained from CO_2_^4–6^, syngas^7^, lignocellulosic biomass^8–10^ or plastic waste^11^. Its biochemical conversion to several value-added products such as glycolate^12^, polyhydroxyalkanoates^13^, or aromatic amino acids (tyrosine, phenylalanine)^14^ has been reported. The assimilation of EG typically proceeds via the intermediate glycolaldehyde. This versatile compound can be assimilated through various pathways, such as the natural tartronic semialdehyde pathway^13,15^, or non-natural acetyl-CoA-yielding routes^16–18^.

In addition, glycolaldehyde is an intermediate of a recently proposed synthetic pathway which enables the conversion of methanol to value-added chemicals via a linear metabolic route^16^. The implementation of linear methanol-assimilating pathways may be a means to render the use of this attractive C_1_ compound more efficient as it avoids the complexity of natural cyclic methanol fixation pathways pertaining to the fixation of formaldehyde by an acceptor molecule (e.g. ribulose-5-phosphate or glycine). The resultant carbon skeleton must be rearranged through a sequence of reactions, with regeneration of the acceptor molecule and the formation of a C_3_ product (e.g. pyruvate or serine), which is then further metabolized in the cell. Any kinetic or stoichiometric imbalance in this chain of reactions can cause methanol assimilation to collapse^19,20^, making it difficult to increase the pathway carbon flux. Thus, glycolaldehyde can be considered to occupy the position of a metabolic hub, from which carbon derived from methanol or ethylene glycol can be efficiently channelled into central metabolism.

In this study, we extend the range of glycolaldehyde-dependent pathways by demonstrating production of 2,4-dihydroxybutyric acid (DHB), which can serve as a precursor for the methionine analogue 2-hydroxy-4-(methylthio)butyrate^21^ or 1,3-propanediol^22^. The non-natural pathway successively employs D-threose aldolase, D-threose dehydrogenase, D-threono-1,4-lactonase, D-threonate dehydratase and 2-oxo-4-hydroxybutyrate (OHB) reductase enzyme activities in the conversion of glycolaldehyde to DHB. Carbon is fully conserved in the sequence of reactions between glycolaldehyde and DHB. The pathway has a 51% higher theoretical yield than its natural counterparts which assimilate glycolaldehyde through tartronic semialdehyde^15^ or β-hydroxyaspartate^23^ and produces DHB through the previously reported malyl-P or malyl-CoA-dependent pathways^21,24^. Following the identification and characterization of candidate enzymes with the requisite catalytic activities, we demonstrate the in vivo conversion of glycolaldehyde to pathway intermediates and the DHB product by step-wise expression of the activities constituting the pathway and ^13^C-based carbon tracing analysis. We show the biosynthesis of 8.2 mM (or 1 g L^−1^) DHB from glycolaldehyde at a yield of 0.11 mol mol^−1^. Extension of the pathway to use of ethylene glycol results in production of 6.75 mM (or 0.8 g L^−1^) DHB from ethylene glycol at a yield of 0.15 mol mol^−1^.

## Results

### Design of the synthetic DHB pathway

**Fig. 1:**
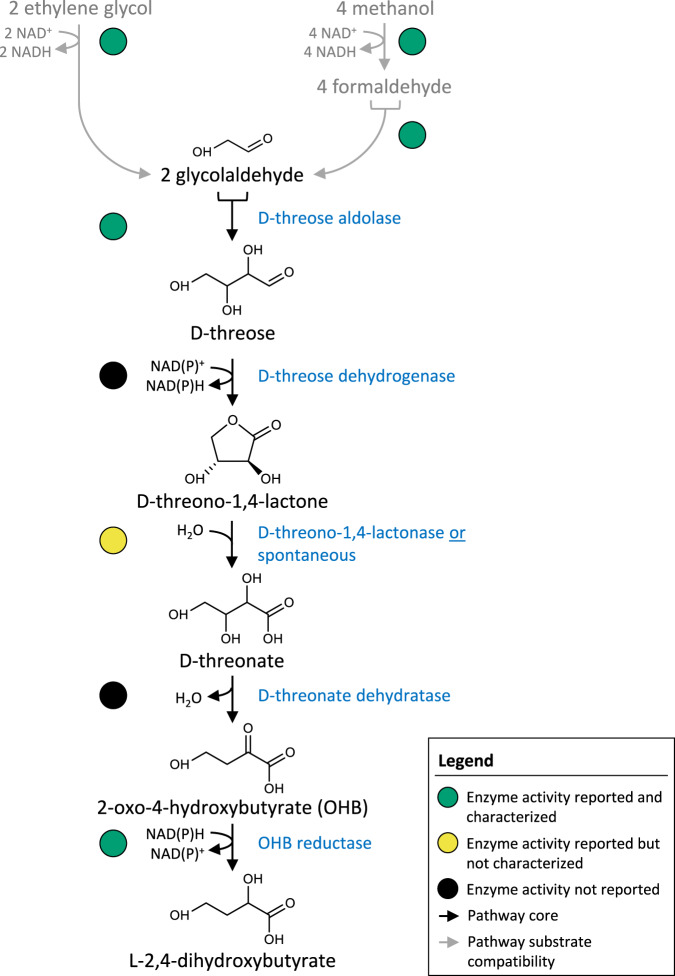
Design of the synthetic DHB pathway. Two molecules of glycolaldehyde are converted without carbon loss into L-2,4-dihydroxybutyrate by five consecutive reaction steps. The pathway is compatible with the use of ethylene glycol or methanol as the starting substrates.

The biosynthesis of the non-natural 2,4-dihydroxybutyric acid (DHB) metabolite from D-glucose and D-xylose sugars has been reported in previous studies^21,24–26^. In this work, we demonstrate the production of DHB from the two-carbon compound glycolaldehyde, which can be biochemically derived either from ethylene glycol^13,15^ or methanol^16^. The conversion of glycolaldehyde to DHB proceeds via five sequential reaction steps (Fig. 1). In the first step, two molecules of glycolaldehyde (GA) are fused by the action of a D-threose aldolase to yield a single D-threose molecule. The resulting four-carbon sugar is oxidized by a D-threose dehydrogenase to yield D-threono-1,4-lactone, which can then be converted to the corresponding sugar acid either spontaneously or in a reaction catalysed by a D-threono-1,4-lactonase. In the last two reaction steps, D-threonate is first dehydrated to 2-oxo-4-hydroxybutyrate (OHB) by a D-threonate dehydratase, and the OHB product is then reduced to yield DHB in a reaction catalysed by an OHB reductase.

The negative standard Gibbs free energy of the proposed pathway starting from glycolaldehyde (−116.2 kJ mol^−1^), ethylene glycol (−92.5 kJ mol^−1^) or methanol (−128.4 kJ mol^−1^) attests to the thermodynamic feasibility of the reaction sequence (see Supplementary Method 1 and Supplementary Table 1). The pathway conserves the carbon between the putative methanol or ethylene glycol substrates and the DHB product, giving rise to a maximum product yield of ~1 g g^−1^ for either substrate (Table 1, Supplementary Figs. 1, 2). The stoichiometry of our pathway respectively confers a 13% and 51% advantage over natural methanol^27^ and ethylene glycol^15,23^ assimilation routes.

**Table 1:** Theoretical maximum yields of 2,4-dihydroxybutyrate (DHB) on methanol (MeOH) and ethylene glycol (EG) according to our synthetic pathway or alternative routes

Subst.	Pathways	Theoretical yield (mol/mol)	Theoretical yield (g/g)	O_2_ demand (mol/mol)
MeOH	RuMP^a^ + malyl-P^b^	0.22	0.83	2.8
	RumP^a^ + malyl-CoA^c^	0.17	0.64	5.0
	Synthetic pathway	0.25	0.94	2.0
EG	Tartronic SA^d^+ malyl-P^b^	0.33	0.64	3.5
	Tartronic SA^d^ + malyl-CoA^c^	0.33	0.64	3.5
	β-hydroxyaspartate cycle^e^ + malyl-P^b^	0.33	0.64	3.5
	β-hydroxyaspartate cycle^e^ + malyl-CoA^c^	0.33	0.64	3.5
	Synthetic pathway	0.50	0.97	1.0

^a^Natural methanol assimilation pathway^27^ via ribulose-5P (RuMP).

^b^Non-natural DHB pathway^21^ derived from L-malate natural precursor via malyl-P intermediate.

^c^Non-natural DHB pathway^24^ derived from glyoxylate/acetyl-CoA natural precursors via malyl-CoA intermediate.

^d^Natural EG assimilation pathway^15^ via tartronic semialdehyde.

^e^EG assimilation pathway via β-hydroxyaspartate cycle which yields oxaloacetate from glyoxylate in 4 reactions^23^.

### Identification of pathway enzymes with known activities

Implementation of the synthetic pathway requires the identification of enzymes possessing the five component catalytic activities. Both D-threose aldolase (TA) and OHB reductase activities have previously been described in the literature^28,29^. The D-fructose-6P aldolase from *E. coli* (Ec.FsaA) has been shown to catalyse the reversible self-aldol addition of glycolaldehyde to yield D-threose^28^. Furthermore, the Ec.FsaA^TA^ (L107Y:A129G) mutant variant was found to be over three orders of magnitude more catalytically efficient for D-threose production than the wild-type enzyme, as determined from the relative values of the respective *V*_*max*_*/K*_*m*_ kinetic parameters^28^. We therefore incorporated this mutant enzyme as a D-threose aldolase in our synthetic pathway. The previously described malate dehydrogenase mutant Ec.Mdh^5Q^, obtained by the introduction of five point mutations in *E. coli* L-malate dehydrogenase (Ec.Mdh I12V:R81A:M85Q:D86S:G179D), is highly active on OHB^29^. Therefore, this enzyme was selected as the OHB reductase (kinetic parameters for which are provided in Table 2) to catalyse the last conversion step in our pathway.

**Table 2:** Kinetic parameters of candidate pathway enzymes used for in vivo studies

Enzyme	Function	*V*_max_ (U mg^−1^)	*K*_*m*_ (mM)	*V*_max_*/K*_*m*_ (U mg^−1^ M^−1^)	Reference
Ec.FucO	EG dehydrogenase	5.7	7	814	Sridhara et al.^59^
Go.Adh	EG dehydrogenase	4.82 (±0.23)^a^	2.40 (±0.19)	2008	Zhang et al.^57^
		7.06 (±0.28)^b^	964 (±84)	0.08	Scheffen et al.^58^
Ec.FsaA^TA c^	D-threose aldolase	5.3 (±0.3)	Donor: 0.094 (±0.008)	2524	Szekrenyi et al.^28^
			Acceptor: 2.1 (±0.3)		
Pc.TadH	D-threose dehydrogenase	3.50 (±0.1)^e^	–	70	This work
Tt.Lac11	D-threono-1,4-lactonase	1.76 (±0.16)	2.92 (±0.13)	603	This work
Hh.AraD	D-threonate dehydratase	0.19 (±0.04)	1.21 (±0.36)	157	This work
Aa.AraD	D-threonate dehydratase	0.12 (±0.06)	1.95 (±0.53)	62	This work
Ec.Mdh^5Q d^	OHB reductase	110.31 (±0.19)	1.6 (±0.01)	68,944	Frazão et al.^29^

^a^Parameters estimated at pH 8.5 (50 mM Tris-HCl) and 0.5 mM NAD^+^. Range of tested EG concentrations was 0.5–20 mM.

^b^Parameters estimated at pH 7.8 (100 mM MOPS-KOH) and 2.0 mM NAD^+^. Range of tested EG concentrations was 10–2000 mM.

^c^Corresponds to double mutant Ec.FsaA L107Y:A129G

^d^Corresponds to quintuple mutant Ec.Mdh I12V:R81A:M85Q:D86S:G179D.

^e^*V*_max_ values refer to the specific activity obtained at 50 mM substrate concentration, since no saturation was observed under tested conditions.

### Screening for missing pathway enzymes

**Fig. 2:**
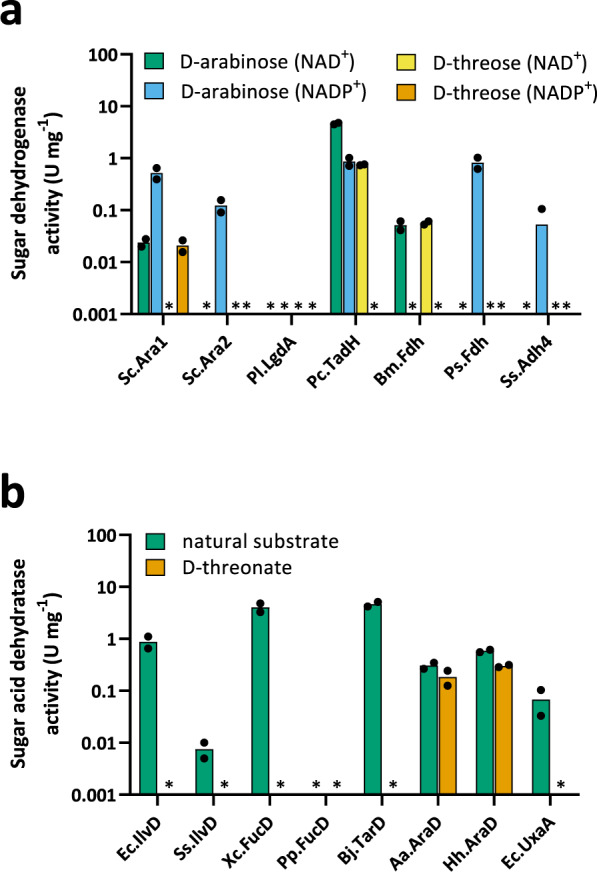
Screening of pathway missing enzymes. Purified N-terminal his-tagged candidate NAD(P)-dependent D-threose dehydrogenase (**a**) and D-threonate dehydratase (**b**) enzymes were tested on natural and synthetic substrates. Absence of measureable activity is indicated by an asterisk (*). Results represent the mean of two biological replicate experiments. Individual data points are shown as coloured black dots. For analysis of candidate D-threose dehydrogenases, substrate concentrations of co-factor and sugar were 10 mM each. All candidate D-threonate dehydratase enzymes were tested on D-threonate and the respective natural substrate (Ec.IlvD, Ss.IlvD: 2R-dihydroxyvalerate; Xc.FucD, Pp.FucD: L-fuconate; Bj.TarD: D-tartrate; Aa.AraD, Hh.AraD: D-arabinonate; Ec.UxaA: D-altronate) using the semicarbazide assay which detects 2-keto acids. Substrate concentrations were 10 mM (except for Aa.AraD and Hh.AraD which were assayed on 1 mM of their natural substrate D-arabinonate). Enzymes with activity on D-threonate were further verified to produce OHB in a coupled assay with OHB reductase Ec.Mdh^5Q^ (see Supplementary Fig. 4). Source data are provided as a Source Data file.

Having selected the D-threose aldolase and OHB reductase pathway enzymes, we set out to identify potential enzymes with the remaining required activities. First, we sought to identify enzymes with D-threose dehydrogenase activity. Sugar dehydrogenases frequently have a pronounced substrate promiscuity which enables them to accept different sugar substrates sharing only limited structural features. In particular, it has been reported that sugar dehydrogenases accept L-glucose, D-arabinose, L-xylose or L-fucose with similar specificity^30–32^. Noting that these sugars possess the same (2S,3R)-hydroxyl group carbon-attachment stereochemical configuration as D-threose, we screened dehydrogenases with confirmed activity on these sugars for additional activity on D-threose. Candidate enzymes included the D-arabinose dehydrogenases from *Saccharomyces cerevisiae* (Sc.Ara1, Sc.Ara2)^33,34^, D-threo-aldose 1-dehydrogenase from *Paraburkholderia caryophylli* (Pc.TadH)^31^, *scyllo*-inositol 2-dehydrogenase from *Paracoccus laeviglucosivorans* (Pl.LgdA)^35^, D-threo-aldose 1-dehydrogenase from *Pseudomonas* sp. 1143 (Ps.Fdh)^36^, D-arabinose dehydrogenase from *Sulfolobus solfataricus* (Ss.Adh4)^37^ and L-fucose dehydrogenase from *Burkholderia multivorans* (Bm.BmulJ04919, herein denoted as Bm.Fdh)^38^. Of the seven candidate enzymes, Sc.Ara1, Pc.TadH and Bm.Fdh displayed measurable activity on D-threose with either NAD^+^ or NADP^+^ co-enzymes (Fig. 2a, Supplementary Table 2). The best enzyme, Pc.TadH, had a maximum specific activity of 0.75 U mg^−1^ (Fig. 2a). No saturation was observed in the presence of concentrations of up to 50 mM of synthetic substrate (Table 2; for detailed analysis on natural substrate, please refer to Supplementary Table 3). Pc.TadH was chosen for final construction of the synthetic pathway.

We next sought to identify an enzyme displaying D-threono-1,4-lactonase activity. Although spontaneous chemical hydrolysis has previously been reported to occur with D-xylonolactone, this is a rather slow process and enzyme-based catalysis significantly accelerates sugar acid formation in vivo^39,40^. Westlake^41^ has reported that the gluconolactonase from *Thermogutta terrifontis* (Tt.Thte1497, herein denoted Tt.Lac11) was able to act on a broad range of sugar-1,4-lactones. The enzyme was also found to be active on D-threono-1,4-lactone, albeit at an unspecified lower rate^41^. We therefore reanalysed the kinetic properties of this enzyme and found comparable catalytic efficiencies for both the natural L-fucono-1,4-lactone and synthetic D-threono-1,4-lactone substrates (see Supplementary Table 4). Since the enzyme also had high affinity towards D-threono-1,4-lactone (*K*_*m*_ = 2.92 mM, see Table 2), it was retained for construction of the synthetic pathway. We note that spontaneous lactone hydrolysis was observed in our assays, although at lower rates than reactions catalysed by Tt.Lac11 (Supplementary Fig. 3).

We finally set out to identify a D-threonate dehydratase enzyme. While dehydratase enzymes with activity on L-threonate have previously been identified^32^, no such enzyme activity on the corresponding D-stereoisomer has been reported to the best of our knowledge. Adopting an analogous strategy to that employed in the identification of D-threose dehydrogenase activities, we selected candidate enzymes with activity on sugar acids with (2S,3R) carbon-centre attachment configuration. These included L-fuconate dehydratases from *Xanthomonas campestris* (Xc.FucD)^42^ and *Pseudomonas putida* (Pp.FucD), D-arabinonate dehydratases from *Acidovorax avenae* (Aa.Acav1654 or ACAV_RS08155, herein denoted as Aa.AraD)^42^ and *Herbaspirillum huttiense* (Hh.E2K99_19880 or C785_RS13685, herein denoted as Hh.AraD)^42^, D-tartrate dehydratase from *Bradyrhizobium japonicum* (Bj.TarD)^43^ and D-altronate dehydratase from *E. coli* (Ec.UxaA)^44^. We additionally included dihydroxy-acid dehydratases from *E. coli* (Ec.IlvD)^45^ and *S. solfataricus* (Ss.IlvD)^32^ in our analysis, of which Ss.IlvD was previously shown to act on a broad range of (2R,3S) and (2S,3R) substrates. Following expression and purification, the enzymes were evaluated for activity on D-threonate using a non-specific assay that detects the 2-keto acid product of the dehydratase reaction via derivatization as the corresponding semicarbazone. Amongst the enzymes tested, Hh.AraD and Aa.AraD displayed significant activity on D-threonate with specific activities of 0.30 and 0.18 U mg^−1^, respectively (see Fig. 2b, Supplementary Table 5). We further confirmed that OHB was the dehydratase reaction product by monitoring NADH-consumption in a coupled enzyme assay system with the OHB-reducing Ec.Mdh^5Q^ enzyme (see Supplementary Fig. 4). When the two dehydratase enzymes were further evaluated, *K*_*m*_ values commensurate with operational functioning of the in vivo pathway (Hh.AraD, 1.21 mM; Aa.AraD, 1.95 mM) were observed (see Table 2; a more detailed analysis is provided in Supplementary Table 6). As a result of this work, all the enzymatic activities required in the implementation of the synthetic metabolic pathway were identified. They were subsequently used to demonstrate in vivo conversion of glycolaldehyde to pathway intermediates and the final DHB product.

### Biosynthesis of D-threonate from glycolaldehyde

Next, we aimed at validation of the in vivo functioning of the synthetic pathway by a stage-wise increase in the number of component enzymes expressed. To further facilitate investigation of pathway behaviour, we decided to split the pathway into two parts: one to produce D-threono-1,4-lactone from glycolaldehyde, and the another to convert D-threonate into DHB. DHB production from glycolaldehyde was finally demonstrated by simultaneous operation of both pathway sections. This was done in the presence and absence of additionally expressed D-threono-1,4-lactonase to clarify the question of whether the pathway could rely on the spontaneous conversion of the lactone to its corresponding linear sugar acid (which is expected to occur at a low rate at neutral pH^39,46^) alone, or whether an enzyme-catalysed reaction step is required.

The *E. coli* TW64 (MG1655 Δ*yqhD* Δ*aldA*) strain was used as the host strain in all experiments. It was primarily selected because it carries deletions in genes that encode aldehyde dehydrogenase (AldA)^47^ and the highly active glycolaldehyde reductase (YqhD)^48^, and presumed therefore to be largely devoid of endogenous enzyme activity able to compete with the conversion of glycolaldehyde to D-threose. Cultivation was carried out in shake flasks containing M9 mineral medium supplemented with LB (10% v/v). While we later show our pathway to function without LB, its addition alleviated strong growth phenotypes observed with cells harbouring high-copy plasmids (see Supplementary Fig. 5). IPTG (0.5 mM) was added when the optical density at 600 nm (OD_600_) of the cultures reached ~0.6 to induce plasmid-borne gene expression. Glycolaldehyde (20 mM) was added when the OD_600_ attained a value of approximately 2.0. Product formation and substrate consumption were estimated by HPLC after 48 h of cell cultivation (see Table 3).

**Table 3:** Whole-cell bioconversion of glycolaldehyde to D-threose and D-threonolactone/D-threonate^a^

Strain	Plasmid	GA cons. (mM)	EG prod. (mM)	D-threose prod. (mM)	D-threonate/lactone ^b^ prod. (mM)	C_4_ yield ^c^ (mol mol^-1^)
TW138	pEXT20	10.40 (±3.41)	1.25 (±0.38)	– ^d^	– ^d^	–
TW145	pEXT20-Ec.fsaA^TA^	7.69 (±0.35)	1.64 (±0.05)	2.31 (±0.57)	– ^d^	0.30 (±0.06)
TW146	pEXT20-Ec.fsaA^TA^-Pc.tadH	8.15 (±0.51)	3.12 (±1.10)	– ^d^	1.10 (±0.20)	0.14 (±0.03)
TW253	pACT3-Ec.fsaA^TA^-Pc.tadH	10.22 (±0.63)	1.05 (±0.05)	– ^d^	3.13 (±0.47)	0.30 (±0.03)

*cons.* consumption, *prod.* production.

^a^*E. coli* TW64 (MG1655 Δ*yqhD* Δ*aldA)* was used as host strain in all experiments. Cells were grown in 250 ml shake flasks containing mineral medium supplemented with LB at 10% (v/v). IPTG (0.5 mM) was added when OD_600_ reached ~0.6, and glycolaldehyde (20 mM) when OD_600_ was ~2.0. Incubation time was 48 h. Results are expressed as mean (±STDV) of at least two biological replicates.

^b^Total amount of D-threonate + D-threonolactone. Both compounds cannot be resolved by our HPLC method but have identical calibration factors linking peak area and concentration.

^c^Corresponds to molar yield of the sum of D-threose + D-threonate/lactone formed divided by glycolaldehyde consumed.

^d^Not detected.

As a first step in the investigation of the biosynthesis of D-threonate from glycolaldehyde, we cloned the candidate D-threose aldolase encoding gene (Ec.*fsaA*_*L107Y:A129G*_) into the high-copy pEXT20 plasmid regulated by the inducible TAC promoter. No D-threose formation was observed in a control experiment using cells containing the empty pEXT20 plasmid (strain TW138). Considerable amounts of glycolaldehyde were consumed (10.4 mM), indicative of the unexpected existence of glycolaldehyde-consuming side reactions. However, in cells expressing the candidate D-threose aldolase enzyme (strain TW145), D-threose was formed (2.31 mM) at a molar yield of 0.30 mol mol^-1^, corresponding to 60% of the theoretical maximum. Encouraged by these results, we continued our analyses by co-expressing the D-threose aldolase Ec.FsaA^TA^ and the D-threose dehydrogenase Pc.TadH from the pEXT20 plasmid. After cultivation of the corresponding TW146 strain in the presence of glycolaldehyde, we observed production of D-threonate/lactone (1.10 mM, Table 3, D-threonate and D-threono-1,4-lactone could not be resolved by our HPLC method), while D-threose could not be detected in the cultivation medium. Expression of these activities from the medium copy pACT3 plasmid further improved pooled D-threonate/lactone formation by ~3-fold (3.13 mM). These results confirmed in vivo functioning of the glycolaldehyde-dependent pathway up to the formation of D-threonate/lactone.

### Biosynthesis of DHB from D-threonate

The two-step conversion of D-threonate to DHB requires the successive application of D-threonate dehydratase and OHB reductase enzymes. Biotransformation of D-threonate, added as a supplement to the culture medium, into DHB can take place only when both enzymatic activities are functionally expressed together with a D-threonate uptake system in the producer strain. However, as far as we are aware, *E. coli* is not capable of D-threonate uptake, rendering the heterologous expression of a D-threonate permease mandatory for the bioconversion of extracellular D-threonate to DHB. A D-threonate importer system, encoded by Re.*kgdT*, has previously been described in *R. eutropha* H16^49^. To test whether this permease could be functionally expressed in *E. coli*, we used a growth-based assay, relying on the heterologous expression of D-threonate kinase and 4-phospho-D-threonate dehydrogenase enzymatic activities, required to convert D-threonate to dihydroxyacetone phosphate, as a means of conferring growth on this compound (Supplementary Fig. 6). Our assay showed that only the strain expressing the permease in addition to the D-threonate-assimilating enzymes was able to grow on D-threonate. This confirmed that Re.KgdT could indeed serve as a D-threonate importer in *E. coli*. We therefore constructed the low-copy pEXT21-Re.kdgT plasmid, which was co-transformed into the *E. coli* TW64 parent strain, together with the compatible low-copy pEXT22 plasmid containing the genes encoding D-threonate dehydratase (Aa.AraD, Hh.AraD) and OHB reductase (Ec.Mdh^5Q^) activities. The resulting strains were cultivated in M9 mineral medium supplemented with LB (10% v/v). IPTG (0.5 mM) and D-threonate (10 mM) were added after 3 h of cultivation. Product formation and D-threonate consumption were measured after 48 h of cultivation (see Table 4). No DHB production was observed in control experiments (strains TW334 and TW336), in which neither the OHB reductase nor the candidate D-threonate dehydratase enzymes were expressed. Only when both OHB reductase and candidate D-threonate dehydratases were co-expressed, was DHB found to be produced with the concomitant consumption of D-threonate. The highest level of DHB production (2.37 mM) was obtained with the strain TW339 expressing the D-threonate dehydratase Hh.AraD. This result demonstrated the in vivo functioning of the pathway segment linking D-threonate to DHB, and identified Hh.AraD as the preferred D-threonate dehydratase enzyme. However, our results showed that only a small fraction of the added D-threonate (4.81 mM) was actually taken up by the cells, and that only part of this was converted into DHB (molar yield of 0.48 mol mol^-1^). This is indicative of slow sugar acid uptake and the presence of unidentified side reactions that divert some of the pathway intermediates into as yet unidentified products.

**Table 4:** Whole-cell bioconversionof D-threonate to DHB ^a^

Strain	Plasmids	D-threonate consumed (mM)	DHB produced (mM)	DHB yield (mol mol^-1^)
TW334	pEXT22/pEXT21	– ^b^	– ^b^	–
TW336	pEXT22/pEXT21-Re.kdgT	– ^b^	– ^b^	–
TW335	pEXT22-Ec.mdh^5Q^/pEXT21-Re.kdgT	– ^b^	– ^b^	–
TW338	pEXT22-Ec.mdh^5Q^-Aa.araD/pEXT21-Re.kdgT	1.51 (±0.49)	0.67 (±0.11)	0.46 (±0.08)
TW339	pEXT22-Ec.mdh^5Q^-Hh.araD/pEXT21-Re.kdgT	4.81 (±0.74)	2.37 (±0.47)	0.48 (±0.02)

^a^*E. coli* TW64 (MG1655 Δ*yqhD* Δ*aldA)* was used as host strain in all experiments. Cells were incubated in 14 mL test tubes containing mineral medium supplemented with LB at 10% (v/v). After 3 h of incubation, IPTG (0.5 mM) and D-threonate (10 mM) were added. Total incubation time was 48 h. Results are expressed as mean (±STDV) of two biological replicates.

^b^Not detected.

### Full pathway synthesis of DHB from glycolaldehyde

To demonstrate biosynthesis of DHB from glycolaldehyde, the entire pathway was simultaneously expressed in the producer strain (see Table 5). Particular attention was paid to the question as to whether or not the expression of a D-threono-1,4-lactonase was necessary given that ring-opening of the lactone may, in principle, also occur spontaneously. To this end, the plasmids pACT3-Ec.fsaA^TA^-Pc.tadH and pEXT22-Ec.mdh^5Q^-Hh.araD were first transformed into the strain *E. coli* TW64, thus, expressing the entire pathway but without the lactonase. The resulting strain (TW290) was capable of accumulating 1.98 mM of D-threonate/lactone after 48 h of cell cultivation. However, DHB production could not be detected. Based on this observation, we hypothesized that no DHB was produced because the spontaneous rate of conversion of the lactone to threonate was too slow. We therefore additionally expressed the D-threono-1,4-lactonase, Tt.Lac11, in strain TW293 to complete the pathway (Fig. 1). However, despite the presence of all pathway enzymes, DHB production was still not observed (Table 5). Only when both the Tt.Lac11 and the D-threonate uptake system Re.KdgT were simultaneously expressed, resulting in strain TW304, DHB was found in the culture broth at a concentration of 0.08 mM (Table 5). We then speculated that rate-limiting intermediate transport steps may impede DHB pathway production and that Tt.Lac11-assisted hydrolysis of the lactone may not occur in the cytoplasm. Indeed, bioinformatic analyses indicated the presence of a signal peptide at the N-terminus of Tt.Lac11 which is characteristic of periplasmic proteins (Supplementary Fig. 7). In an attempt to avoid the need to export and then reimport the D-threono-1,4-lactone and D-threonate pathway intermediates, we therefore created three truncated forms of Tt.Lac11 with variable N-terminal sections of the sequence, including the signal peptide-exporting sequence, removed (please refer to Supplementary Methods 2 and 3 for a more complete description). Notably, expression levels of the lactonase variant Tt.Lac11^v1^ (Δ1-38 aa) in *E. coli* were improved 34-fold, and the specific activity on D-threono-1,4-lactone was increased by 10-fold (see Supplementary Table 7). Hence, this variant was chosen to provide the necessary cytoplasmic lactonase activity (see Supplementary Figs. 8, 9) in the synthetic pathway. Strains expressing the improved lactonase (TW354 and TW356) accumulated increased levels of DHB from glycolaldehyde (see Table 5). However, the highest DHB titre (0.16 mM) was obtained with strain TW354, in which the Re.kdgT D-threonate importer system was still expressed. We observed identical trends when aiming at DHB production from D-threose (Supplementary Fig. 8, Supplementary Method 2), suggesting that lactonase expression levels are likely to be limiting and that D-threono-1,4-lactone continues to be secreted by the cells.

**Table 5:** Whole-cell bioconversion of glycolaldehyde to DHB^a^

Strain	Plasmids	GA cons. (mM)	EG prod. (mM)	D-threose prod. (mM)	D-threonate/ lactone prod. (mM)	DHB prod. (mM)
TW288	pACT3-Ec.fsaA^TA^-Pc.tadH/pEXT22	17.56 (±1.33)	1.10 (±0.25)	0.14 (±0.13)	2.63 (±0.39)	– ^b^
TW290	pACT3-Ec.fsaA^TA^-Pc.tadH/pEXT22-Ec.mdh^5Q^-Hh.araD	18.75 (±5.57)	0.32 (±0.27)	0.76 (±0.08)	1.98 (±0.06)	– ^b^
TW293	pACT3-Ec.fsaA^TA^-Pc.tadH-Tt.lac11/pEXT22-Ec.mdh^5Q^-Hh.araD	14.02 (±2.33)	0.84 (±0.01)	0.20 (±0.09)	2.69 (±0.18)	– ^b^
TW304	pACT3-Ec.fsaA^TA^-Pc.tadH-Tt.lac11/pEXT22-Ec.mdh^5Q^-Hh.araD /pEXT21-Re.kdgT	12.77 (±6.41)	0.48 (±0.15)	1.59 (±0.19)	2.39 (±1.59)	0.08
TW354	pACT3-Ec.fsaA^TA^-Pc.tadH-Tt.lac11^v1^/pEXT22-Ec.mdh^5Q^-Hh.araD /pEXT21-Re.kdgT	15.61 (±2.44)	0.30 (±0.04)	2.99 (±0.41)	1.41 (±0.14)	0.16 (±0.04)
TW356	pACT3-Ec.fsaA^TA^-Pc.tadH-Tt.lac11^v1^/pEXT22-Ec.mdh^5Q^-Hh.araD	16.29 (±0.27)	0.70 (±0.21)	1.37 (±0.34)	1.80 (±0.33)	0.10 (±0.005)

^a^*E. coli* TW64 (MG1655 Δ*yqhD* Δ*aldA)* was used as host strain in all experiments. Cells were grown in 250 mL shake flasks containing mineral medium supplemented with LB at 10% (v/v). IPTG (0.5 mM) was added when OD_600_ reached ~0.6, and glycolaldehyde (20 mM) when OD_600_ was ~2.0. Incubation time was 48 h. Results are expressed as mean (±STDV) of two biological replicates.

^b^Not detected.

### Optimization of DHB production from glycolaldehyde

**Fig. 3:**
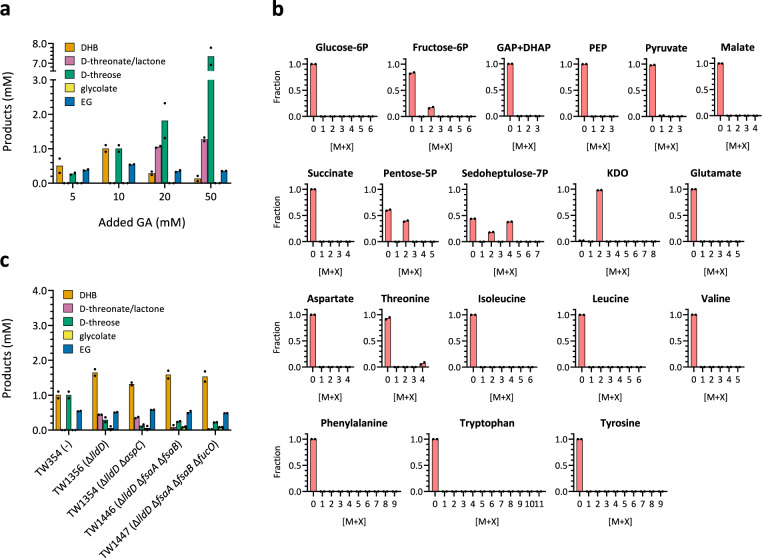
Optimization of DHB biosynthesis in shake-flask experiments. **a** Influence of glycolaldehyde supplementation on DHB production after 24 h of cell cultivation using strain TW354. **b** Labelling pattern of selected metabolites during whole-cell bioconversion experiments of [1,2−^13^C_2_] glycolaldehyde (10 mM) to DHB employing strain *E. coli* strain TW354. Sample for intracellular metabolites was withdrawn 1 h after feeding labelled glycolaldehyde. With exception of KDO, retention times of all shown analytes were verified by injecting unlabelled standards. **c** Host strain engineering for improving DHB production. Initial glycolaldehyde concentration was 10 mM. Total incubation time was 24 h. All strains are derived from TW354 (MG1655 Δ*yqhD* Δ*aldA*/pACT3-Ec.fsaA^TA^-Pc.tadH-Tt.lac11^v1^/pEXT22-Ec.mdh^5Q^-Hh.araD/pEXT21-Re.kdgT), and additional chromosomal modifications are indicated in the X-axis between parentheses. Cells were grown in mineral medium supplemented with LB at 10% (v/v). IPTG (0.5 mM) was added when OD_600_ reached ~0.6, and glycolaldehyde when OD_600_ was ~2.0. Results represent the mean of two biological replicate experiments. Individual data points are shown as coloured black dots. Legend: DHAP, dihydroxyacetone-P; GAP, glyceraldehyde-3P; KDO, 3-deoxy-α-D-manno-2-octulosonate; PEP, phosphoenolpyruvate. Source data are provided as a Source Data file.

Next, we sought to optimize DHB production from glycolaldehyde (Fig. 3) and found that the concentration of added substrate had a major impact on the product spectrum which was monitored after 24 h of incubation (Fig. 3a). While lower substrate concentrations resulted in DHB being the major product of the pathway, supplementation of higher glycolaldehyde amounts led to increased accumulation of D-threose and a severe drop of DHB concentration. Since the highest DHB titre of 1 mM was found when feeding cells with 10 mM glycolaldehyde, we retained this condition for further investigations.

To confirm that the DHB produced in the biotransformation experiments was indeed derived from glycolaldehyde and to identify reactions which had deviated carbon away from the synthetic pathway, we performed ^13^C-metabolite tracer experiments. We fed cultures of strain TW354 with 10 mM fully labelled [1,2-^13^C_2_] glycolaldehyde under otherwise identical cultivation conditions, and recorded the labelling pattern of the final DHB product and of key intermediates in the central carbon and amino acid metabolism of the cells using LC/MS. As shown in Supplementary Fig. 10, ^13^C labelling of DHB unambiguously demonstrates the successful in vivo operation of the synthetic pathway. Quantitatively, 99.8% of the isotope in the fully labelled ^13^C-glycolaldehyde pathway substrate was retained in the fully labelled DHB (M + 4).

Since cells went through a transient growth arrest after exposure to glycolaldehyde (Supplementary Fig. 11), metabolic steady state could not be established. Therefore, these isotopologue measurements could not be used to derive an actual metabolic flux map, but provide rather qualitative information about the pathways implicated in glycolaldehyde assimilation. We found 7% of the intracellular threonine to contain four labelled carbon atoms (M + 4) (Fig. 3b; no threonine was found in the supernatant). Since aspartate did not contain any labelled carbon, this indicated that the ^13^C-labelled threonine fraction was not produced by the natural aspartate-dependent pathway but derived from OHB via homoserine instead (Supplementary Fig. 12). We speculated that loss of OHB via this unwanted pathway could be reduced by lowering its intracellular concentration, thus rendering any homoserine-yielding transaminations thermodynamically less favourable, and/or by deleting enzymes bearing homoserine aminotransferase activity. Therefore, we deleted the membrane bound L-lactate dehydrogenase (LldD) which was previously shown to oxidise DHB into OHB^22^ thus potentially decreasing the intracellular concentration of the latter. The resulting strain TW1356 accumulated 1.65 mM DHB after 24 h of cultivation which corresponded to an increase of the product titre of 65% compared to the TW354 parental strain (Fig. 3c). The additional deletion of the aspartate transaminase AspC, previously applied in the conversion of homoserine to OHB^26^ did not provide a further increase of the DHB yield (Fig. 3c), presumably because several other endogenous transaminases (including IlvE, TyrB and AlaC^26,50^) possess background homoserine aminotransferase activity, all of which could not be deleted without severely compromising growth of the producer strain.

In addition, we found that nearly 100% of the cell wall precursor 3-deoxy-α-D-manno-2-oculosonate^51^ (KDO, metabolite identified based on its mass due to unavailable standard) contained two labelled carbon atoms (M + 2, Fig. 3b), indicating that its precursor arabinose-5P (Ara5P) was exclusively synthesized from glyceraldehyde-3P and glycolaldehyde in a reaction potentially catalysed by endogenous FsaA and FsaB enzymes previously shown to bear Ara5P aldolase activity^52,53^. From the presence of labelled carbon in the pentose-5P sugar pool (40% M + 2; Ara5P, xylulose-5P, ribulose-5P, and ribose-5P could not be resolved by our LC/MS method), in fructose-6P (17% M + 2), and in sedoheptulose-7P (18% M + 2, 38% M + 4, Fig. 3b) we concluded that glycolaldehyde-derived carbon had been also diverted away from the target pathway by the joint action of Ara5P isomerase, transketolase and transaldolase activities (Supplementary Fig. 12). However, deletion of the endogenous FsaA and FsaB activities did not lead to any improvements of the DHB titre (Fig. 3c). This finding indicated that either the significant residual wild-type activity of the Ec.Fsa^TA^ mutant^28^ was responsible for Ara5P formation, and/or that the loss of carbon through these reactions was not significant (no labelled carbon was found in the amino acids tryptophan, tyrosine and phenylalanine, which are derived from the pentose phosphate pathway intermediate erythrose-4P, see Supplementary Fig. 12). Finally, ^13^C-analyses revealed that glycolaldehyde-derived carbon was not lost through the TCA cycle in the form of CO_2_, which could be inferred from the (near) complete absence of ^13^C carbon in pyruvate and glutamate. Additional deletion of glycolaldehyde-accepting reductase (FucO)^54^ did not yield improved results.

### Bioreactor cultivation of DHB-producing strain

**Fig. 4:**
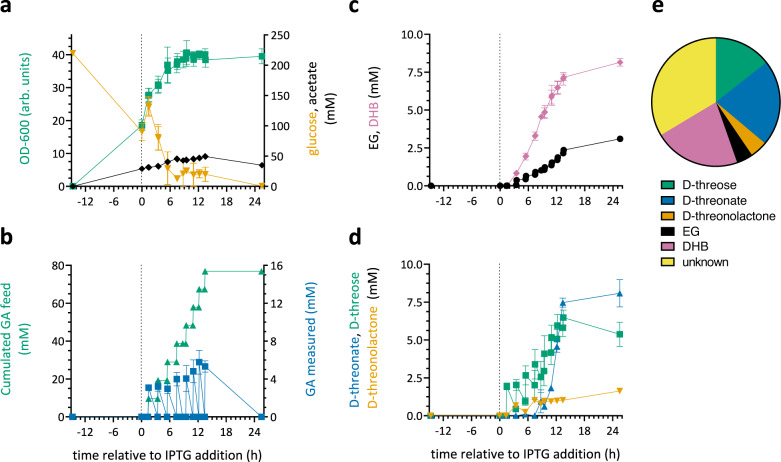
Fermentation profile of DHB-producing strain TW1356. The strain was cultivated in defined mineral medium supplemented with glucose for growth. The working volume of the bioreactor was 1 L (**a**). At 1.5 h after pathway induction by addition of IPTG (0.5 mM), repeated addition of 10 mM glycolaldehyde (GA) was started at intervals of ~2 h (**b**) and product formation was monitored (**c**, **d**). A balance for GA-derived carbon is shown for the end of the fed-batch fermentation at 24 h (**e**). Results are the mean of two biological replicate experiments. Error bars correspond to the standard deviation of the mean. Source data are provided as a Source Data file.

DHB production by strain TW1356 was further investigated in bioreactor cultivations (Fig. 4) where the pH was fixed at 7 and aerobic conditions were assured by preventing dissolved oxygen concentration to fall below 30%. The strain was grown on glucose-containing defined mineral medium to a cell density of ~10 g CDW L^−1^ (corresponding to an OD_600_ value of 18.5) before expression of DHB pathway enzymes was induced by addition of 0.5 mM IPTG (Fig. 4a). Starting 1.5 h after pathway induction, glycolaldehyde was repeatedly fed to the culture in a pulse-wise manner preventing its concentration to exceed 10 mM at the beginning of each feeding cycle. Intervals between glycolaldehyde additions were approximately 2 h allowing for near complete consumption of the aldehyde, thus avoiding accumulation of glycolaldehyde concentrations that were deleterious for DHB production (see Fig. 4b). In the first 10 h after IPTG induction, 4.8 mM of DHB were produced from 40 mmol glycolaldehyde corresponding to a yield of 0.13 mol mol^−1^ (or 0.26 g g^−1^) (Fig. 4c). During this cultivation phase, D-threose and EG were the major identified by-products. At later stages of the cultivation, DHB accumulation continued until reaching a maximum of 8.2 mM (1 g L^-1^) after 24 h. However, the overall DHB yield slightly dropped to 0.11 mol mol^−1^ (0.22 g g^−1^) (Fig. 4c, d). The resolution of D-threonolactone and D-threonate by the colorimetric hydroxamate method^55,56^ showed that the latter compound was the major fermentative by-product accumulating in the medium (8.1 mM) (Fig. 4d). Accumulation of D-threonate started with the transition of the culture into stationary phase at approx. 7 h after IPTG induction, indicating that the dehydratase enzyme is rapidly inactivated in resting cells. The reasons for this behaviour are not clear. In any case, our results indicate that DHB can be produced from glycolaldehyde at significant yields, which could be further increased if the carbon from the pathway intermediates D-threose and D-threonate/lactone is further converted to DHB by employing improved pathway enzymes. In addition, a balance reveals that only 66% of the carbon initially added in the form of glycolaldehyde was recovered in the form of DHB pathway intermediates and ethylene glycol (Fig. 4e). Thus, a significant portion of glycolaldehyde and/or DHB pathway intermediates was deviated from the pathway, possibly, by reactions discussed in the previous section.

### Extension of DHB pathway to the utilization of ethylene glycol

The proposed pathway is intended for the conversion of the low-cost methanol or ethylene glycol alcohols into DHB. To demonstrate the feasibility of this approach, we extended our pathway to the direct bioconversion of ethylene glycol into DHB by the addition of an ethylene glycol oxidation step (Fig. 1). Several NAD(P)^+^-dependent ethylene glycol dehydrogenases have been reported in the literature, including the NAD^+^-dependent alcohol dehydrogenase from *Gluconobacter oxydans* (Go.Gox0313, herein denoted Go.Adh)^57,58^, the L-1,2-propanediol oxidoreductase from *E. coli* (Ec.FucO)^59^ and the oxygen-insensitive Ec.FucO I6L:L7V variant^60^. We first expressed candidate enzymes in the pEXT20 high-copy plasmid using *E. coli* TW64 as the host strain, and evaluated their ethylene glycol dehydrogenase activities (50 mM substrate). While we observed no activity with protein extracts from cells containing an empty plasmid, all candidate enzymes were active on EG (Supplementary Table 8). To evaluate the potential of the different NAD^+^-dependent enzymes to serve in in vivo applications, we developed a growth-based assay using EG as the sole carbon source (see Supplementary Fig. 13). When the candidate enzymes were expressed in a strain devoid of the YqhD glycolaldehyde reductase, expression of Go.Adh yielded the fastest cell growth. This enzyme was therefore selected for inclusion to complete the synthetic pathway.

**Fig. 5:**
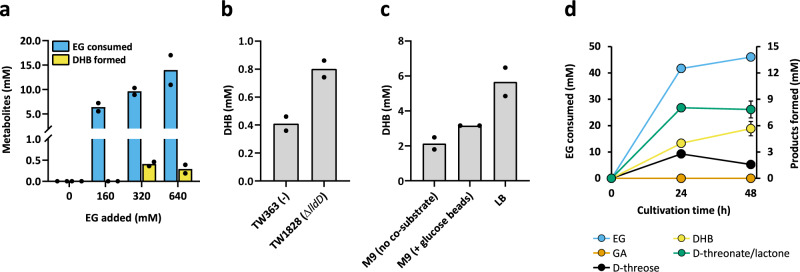
Bioconversion of EG to DHB. Results are shown for incubation times of 48 h using growing (**a**, **b**) and resting cells (**c**, **d**). **a** Impact of EG concentrations on DHB biosynthesis by exponentially growing cells of strain TW363 (derivative of MG1655 Δ*yqhD* Δ*aldA*). **b** Impact of deleting the DHB dehydrogenase LldD on EG to DHB conversion. EG was added at a final concentration equal to 320 mM. **a**, **b** Exponentially growing cells of strains TW363 and TW1828 (derivative of MG1655 Δ*yqhD* Δ*aldA* Δ*lldD*) were grown at 37 °C, 220 rpm in 25 mL mineral medium supplemented with LB at 10% (v/v) with a starting OD = 0.2. IPTG (0.5 mM) and EG were added when OD_600_ reached ~0.6. Results represent the mean of two biological replicate experiments. Individual data points are shown as coloured black dots. **c** Impact of medium composition on conversion of 320 mM EG to DHB using resting cells of strain TW1828. Fermentation profile of EG bioconversion in LB medium is shown in (**d**). **c**, **d** Cells were grown in 200 mL mineral medium supplemented with LB at 10% (v/v) with a starting OD = 0.2. IPTG (0.5 mM) was added when OD_600_ reached ~0.6. After 6.5 h, cells were concentrated to OD_600_ = 15 in 5 mL of indicated medium containing EG and IPTG (0.5 mM) and incubated at 37 °C, 220 rpm. Where indicated, addition of one glucose feed-bead was carried to facilitate the controlled release of the sugar (with a feed rate of ~0.5 mM or 0.09 g L^−1^ per hour, corresponding to a total added concentration of 25 mM or 4.5 g L^−1^). Results are expressed as mean of two biological replicates. Error bars represent the standard deviation of the mean. Source data are provided as a Source Data file.

We then set out to demonstrate direct DHB formation from ethylene glycol by co-expressing all required enzymatic activities in a single strain (Fig. 5). To this end, we constructed the plasmid pACT3-Go.adh-Ec.fsaA^TA^-Pc.tadH-Tt.lac11^v1^ which was co-transformed with pEXT22-Ec.mdh^5Q^-Hh.araD and pEXT21-Re.kdgT into the *E.coli* TW64 host strain. The resulting TW363 strain was cultivated in M9 minimal medium supplemented with LB (10% v/v). IPTG (0.5 mM) and differing amounts of ethylene glycol were added at OD_600_ ~0.6. In control experiments in which ethylene glycol was not added, no DHB production was observed after 48 h of cell cultivation (Fig. 5a). Only in the presence of ethylene glycol were cells able to produce DHB. Maximum DHB production (0.41 mM) was observed in the presence of 320 mM (~20 g L^−1^) ethylene glycol (Fig. 5a). We then confirmed the positive effect of the LldD deletion by constructing strain TW1828 which was able to produce up to 0.80 mM DHB under these conditions (Fig. 5b) and found that DHB production was highest when cultivating the strains in rich LB medium free of glucose (Supplementary Table 9). In an attempt to further increase DHB titres, IPTG-induced pre-cultures of strain TW1828 were concentrated to OD_600_ = 15 in various media containing EG (320 mM) (Fig. 5c). We found LB as the most suited medium to reach higher DHB concentrations (5.66 mM). Again, DHB production was accompanied by accumulation of pathway intermediates at relevant amounts (notably D-threonate/lactone) (Fig. 5d). Because Hh.AraD is a Fe-S dependent dehydratase and Fe-S cluster biogenesis may be limiting inside cells, we additionally supplemented the LB medium with small amounts of L-cysteine and FeCl_3_, but despite a 19% increase in DHB production (6.75 mM), no strong decrease in D-threonate/lactone accumulation was achieved (Supplementary Table 9).

## Discussion

It is possible to couple biochemical product synthesis to the use of CO_2_ via the initial (electro)chemical production of methanol^61^ or ethylene glycol^4–6^, and the subsequent utilization of these intermediates as substrates in fermentation processes^13,16^. Biochemical pathways for the assimilation of both compounds converge at the level of glycolaldehyde, which can be considered as a metabolic hub from where carbon can be further channelled into different metabolic routes. Linear biosynthetic pathways are often preferred over cyclic pathways that require regeneration of acceptor molecules, as their capacity can be conveniently increased by the overexpression of constitutive enzymes. Our work extends the spectrum of linear glycolaldehyde-dependent pathways by demonstrating the feasibility of producing the C_4_ molecule DHB via a five-step synthetic metabolic pathway.

We have successfully demonstrated the biosynthesis of DHB from glycolaldehyde and ethylene glycol, albeit at low rates and titres. The efficiency of DHB production is reduced for several reasons. The unfavourable thermodynamic equilibrium of the initial ethylene glycol oxidation step limits glycolaldehyde accumulation, and therefore flux through the subsequent reaction steps. Incorporation of the pathway into microorganisms that employ irreversible pyrroloquinoline quinone-dependent ethylene glycol dehydrogenases (e.g. *Pseudomonas putida*^13^), combined with the engineering of D-threose aldolase and D-threose dehydrogenase enzymes with higher substrate affinity may help to address this problem. Furthermore, some of the pathway enzymes, including the D-threonate dehydratase (Hh.AraD), exhibit low turnover numbers, thus limiting carbon flux. In addition, accumulation of D-threonate in the supernatant upon transition of growing cells into stationary phase indicates that the D-threonate dehydratase enzyme quickly loses activity under these conditions. Stabilizing the dehydratase activity by either finding a more stable enzyme or by maintaining at least residual growth of the cells appears to be mandatory for further improving pathway performance.

It remains unclear why expression of a D-threonate importer is necessary to increase flux through the pathway. However, this finding could be explained by the fact that D-threono-1,4-lactonase activity levels are insufficient to avoid D-threono-1,4-lactone export. Finally, the observation that the carbon yield of the pathway does not reach its maximum value even with the addition of the D-threonate intermediate suggests that unidentified metabolic side reactions may be operating which divert carbon elsewhere, away from the target pathway.

While further enzyme and metabolic engineering efforts are required to improve the functional efficiency of this pathway, our work does provide an attractive means with which to convert the sustainable carbon source ethylene glycol (and potentially methanol) into an added-value compound of considerable industrial interest. Our study contributes to the increasing capabilities of biochemical engineering in the design and implementation of synthetic reaction sequences with favourable stoichiometries, which are expected to lead to more efficient (bio)chemical routes for the utilization of CO_2_.

## Methods

### Chemicals and reagents

All chemicals and solvents were purchased from Sigma-Aldrich (St. Louis, MO, USA) unless otherwise stated. [1,2-^13^C_2_] Glycolaldehyde (enrichment, 99%) was obtained from Omicron Biochemicals (South Bend, IN, USA). Restriction endonucleases and DNA-modifying enzymes were purchased from New England Biolabs (Ipswich, MA, USA) and used according to manufacturer’s instructions. DNA plasmid isolation was performed using Monarch Plasmid Miniprep Kit (New England Biolabs). DNA extraction from agarose gel and purification of PCR products were carried out using the Monarch DNA Gel Extraction Kit (New England Biolabs). DNA sequencing was carried out by Eurofins SAS (Ebersberg, Germany).

### Protein cloning, expression and purification

*E. coli* DH5α (New England Biolabs) was routinely used for construction of plasmids. All candidate D-threose dehydrogenase, D-threono-1,4-lactonase or D-threonate dehydratase enzymes used in this study are listed in Supplementary Table 10. Genomic DNAs were purchased from DSMZ (Braunschweig, Germany) and served as preferential template for PCR reactions. In their unavailability, synthetic genes (sequences available from Supplementary Data 1) were ordered from Eurofins SAS or Genewiz (Leipzig, Germany) and gene codon-optimization was performed whenever multiple rare codons (assessed as relative adaptness <10%) were detected in natural DNA sequences (https://gcua.schoedl.de/). Genes were amplified by PCR and cloned into the corresponding sites of pET28a (Novagen®, Merck, Darmstadt, Germany) as detailed in Supplementary Data 2, with the incorporation of a N-/C-terminal hexa-His tag. Resulting plasmids were transformed into competent *E. coli* DH5α cells, from which they were extracted and validated by diagnostic PCR and subsequent DNA sequencing as carrying the desired inserts.

Enzymes were expressed in *E. coli* BL21(DE3) cells (New England Biolabs). After an initial screening round to establish optimal expression conditions, proteins were expressed in 50 mL of either lysogeny broth (LB, at 37 °C or 16 °C) or auto-induction medium (at 25 °C) (for details, see Supplementary Table 11). In both cases, cultures were supplemented with 50 µg mL^−1^ kanamycin. After appropriate incubation times, cells were harvested by centrifugation (15 min at 1664 *g*, 4 °C) and pellets were stored at -20 °C until further analysis. Protein purification by immobilized metal affinity chromatography was carried out based on the previous work of Walther et al.^21^. Briefly, frozen cell pellets were thawed on ice, resuspended in 1 mL of ice-cold lysis buffer (50 mM Hepes, 300 mM NaCl, pH 7.5) and broken open by four successive rounds of sonication (sonication interval: 30 s, power output: 40%, UDS 751, Topas, Dresden, Germany). Cell debris was removed by centrifuging crude extracts at 15,493 g for 15 min, 4 °C. The resulting supernatant was incubated for 20 min at room temperature with 0.6 mL of Talon^TM^ Cobalt affinity resin (Cytiva, Marlborough, MA, USA), which was pre-washed twice with 3 mL of lysis buffer. The suspension was centrifuged at 700 *g* for 5 min, 4 °C and resin washed once with 3 mL of lysis buffer. After a new round of centrifugation, the resin was washed with 3 mL of wash buffer (50 mM Hepes, 300 mM NaCl, 15 mM imidazole, pH 7.5) before his-tagged enzymes were eluted with 0.5 mL of elution buffer (50 mM Hepes, 300 mM NaCl, 250 mM imidazole, pH 7.5).

### Enzyme assays

Protein concentrations were determined prior to enzyme assays by the method of Bradford (Roti^©^-Quant, Carl Roth, Karlsruhe, Germany). A detailed description of all enzyme activities measured in this work is provided in Supplementary Method 4. All enzyme assays were carried out in a TECAN Infinite M200pro device (TECAN, Männedorf, Switzerland) using I-control software (version 3.8.2.0, TECAN) for data collection. Unless otherwise stated, all assays were performed for 20 min at 37 °C in 96-well flat bottomed microtiter plates in a final volume of 250 µL. Maximum reaction velocity (*V*_max_) and Michaelis constant (*K*_*m*_) parameter values were estimated by non-linear regression fitting of kinetic data obtained at a minimum of five different substrate concentrations to the Michaelis-Menten equation using Matlab software (version R2018b, Mathworks Inc., Natick, MA, USA).

### Plasmid and strain construction for pathway operation

All plasmids and strains constructed and used in this study are listed in Supplementary Tables 12 and 13, respectively. *E. coli* MG1655 Δ*yqhD* Δ*aldA* was used as the parental strain throughout this study. Additional deletions were introduced by phage transduction^62^ using phage lysates prepared from single-deletion donor strains of the KEIO collection^63^. Positive clones were selected on LB agar plates containing kanamycin (50 µg mL^-1^) and verified by PCR analysis. The kanamycin cassette was removed from the genome by expressing FLP recombinase from the pCP20 plasmid^64^ and correct excision of the cassette was verified by PCR using locus specific primers (Supplementary Data 3). Plasmid-based expression was achieved by integrating the desired gene sequence(s) into appropriate expression vectors following PCR-restriction cloning (for a detailed description, see Supplementary Method 5). In total, four compatible expression vectors^65^ were used: pEXT20 (ColE1 origin, ampicillin resistance), pACT3 (p15A origin, chloramphenicol resistance), pEXT22 (IncFII origin, kanamycin resistance) and pEXT21 (IncW origin, spectinomycin resistance). In all vectors, gene expression was under control of the IPTG-inducible TAC promoter. All primers used for cloning introduced unique restriction sites flanking the gene of interest. Forward primers contained a ribosome binding sequence (RBS: 5’-GTTTAACTTTAAGAAGGAGATATACC-3’) immediately upstream of the coding sequence. The primers used are listed in Supplementary Data 3. All resulting constructions were transformed into DH5α chemically competent *E. coli* cells and verified by diagnostic PCR/restriction analysis and subsequent DNA sequencing as containing the correct insert(s). Plasmids were then transformed in target *E. coli* strains using standard protocols^66^.

### Shake flask whole-cell bioconversions

All cell cultivation was carried out at 37 °C on a rotary shaker (Infors HT, Bottmingen, Switzerland) running at 220 rpm. Pre-cultures were grown in 5 mL of LB in 50 mL falcon tubes. After ~10 h, 500 µL of these cultures were used to inoculate a second pre-culture (10 mL of 90% v/v M9 mineral medium and 10% v/v LB in 50 mL falcon tubes) which was cultivated overnight. The necessary biomass to start main cultures with a starting OD_600_ of 0.2 was transferred to medium composed of 90% (v/v) M9 mineral medium and 10% (v/v) LB. Optical densities were measured using a GENESYS 150 UV-Vis spectrophotometer (ThermoFisher Scientific, Waltham, MA, USA). IPTG (0.5 mM) was added when the OD_600_ reached ~ 0.6. Precursor molecules (^13^C-labelled glycolaldehyde, or unlabelled glycolaldehyde, ethylene glycol or D-threonate) were then added. Briefly, glycolaldehyde was supplied at OD_600_ ~ 2, while ethylene glycol or D-threonate were added when the OD_600_ reached ~ 0.6. Antibiotics were added when required at standard concentrations (chloramphenicol, 35 µg mL^-1^; kanamycin, 50 µg mL^-1^; ampicillin, 100 µg mL^-1^; spectinomycin, 100 µg mL^-1^). One litre of M9 mineral medium contained: 20 g glucose, 18 g Na_2_HPO_4_*12H_2_O, 3 g KH_2_PO_4_, 0.5 g NaCl, 2 g NH_4_Cl, 0.5 g MgSO_4_*7H_2_O, 0.015 g CaCl_2_*2H_2_O, 1 mL of 0.06 M FeCl_3_ stock solution (prepared in 100-fold diluted concentrated HCl), 2 mL of 10 mM thiamine HCl stock solution, 20 g MOPS, and 1 mL of trace element solution (containing per litre: 0.4 g Na_2_EDTA*2H_2_O, 1.8 g CoCl_2_*6H_2_O, 1.8 g ZnSO_4_*7H_2_O, 0.4 g Na_2_MoO_4_*2H_2_O, 0.1 g H_3_BO_3_, 1.2 g MnSO_4_*H2O, 1.2 g CuCl_2_*H_2_O). The pH of the medium was adjusted to 7.0 prior to filter-sterilization.

### Bioconversion of EG using resting cells

Pre-cultures were grown in 5 mL of LB in 50 mL falcon tubes. After an overnight incubation, the necessary biomass to start cultures with a starting OD_600_ of 0.2 was transferred to 200 mL medium composed of 90% (v/v) M9 mineral medium and 10% (v/v) LB. IPTG at a concentration of 0.5 mM was added when the OD_600_ reached ~ 0.6 and cells were incubated for an additional 6.5 h period to allow for protein expression. Cells were then harvested by centrifugation (4,500 g for 10 min at 20 °C) and used to inoculate 5 mL of medium supplemented with EG with OD_600_ = 15. Three basic medium compositions were tested: (1) M9 (no co-substrate), which corresponds to M9 mineral medium without glucose; (2) M9 (glucose beads), referring to M9 mineral medium in which freely dissolved glucose was replaced with 1 glucose FeedBead® (12 mm polymer disk, Adolf Kühner AG, Basel, Switzerland) which allows for a slow, controlled release of glucose (25 mM after 48 h at a rate of ~0.5 mM h^-1^); and (3) LB medium. All media contained 320 mM EG. When required, media were further supplemented with L-cysteine hydrochloride (1 mM) and FeCl_3_ (0.06 mM). Antibiotics were added at standard concentrations.

### Bioreactor whole-cell bioconversion

A single colony was used to inoculate 10 mL of LB containing appropriate antibiotics. After 8 h of cell cultivation, 50 mL of M9 mineral medium were inoculated with 1:500 of first pre-culture. Cells were harvested after 20 h of cultivation, washed once with sterile water and used to inoculate a 3.6 L bioreactor (Labfors 5, Infors HT) that initially contained 1 L of fermentation medium with a starting OD_600_ of ~0.05. The culture condition was set at 37 °C and pH 7.0. Fermentation media for bioreactor cultures contained, per litre: 2 g Na_2_HPO_4_*12H_2_O, 0.8 g KH_2_PO_4_, 6 g (NH_4_)_2_HPO_4_, 0.4 g (NH_4_)_2_SO_4_, 2 g NH_4_Cl, 0.5 g NaCl, 0.5 g MgSO_4_*7H_2_O, 0.015 g CaCl_2_*2H_2_O, 1 mL of 0.06 M FeCl_3_ stock solution, 2 mL of 10 mM thiamine HCl stock solution, and 2 mL of trace element solution (as indicated above). Initial glucose concentration was adjusted to 40 g L^-1^. The pH was controlled at 7.0 by automatic addition of 12.5% NH_4_OH, and the reactors were aerated with air at 0.25-1 vvm. Dissolved oxygen concentration was maintained above 30% with respect to air saturation by automatically raising stirrer speed (400 to 1500 rpm) and aeration rate. Struktol® J 673 (Schill + Seilacher, Hamburg, Germany) was used as antifoaming agent during the culture. Expression of proteins was induced with 0.5 mM IPTG after 15 h (OD_600_ ~ 18.5) and the first glycolaldehyde spike (10 mM) was performed 1.5 h after induction. Additional glycolaldehyde was spiked at a final concentration of 10 mM each time cell recovery was detected by a raising oxygen uptake rate (OUR), which was measured using the O_2_ and CO_2_ content of the exhaust gas (Easy Line EL3020, ABB Automation GmbH, Zurich, Switzerland). Glucose stock solution (400 g L^-1^) was manually added with the first glycolaldehyde spike and whenever glucose limitation led to a drop of the OUR.

### Analysis of extracellular metabolites

All samples were centrifuged (2 min at 11,300 g), syringe-filtered (pore size, 0.2 µm), and kept at -20 °C until analysis. The extracellular concentrations of sugars, aldehydes, alcohols and organic acids were measured on a Smartline series HPLC system (Knauer, Berlin, Germany) equipped with a RI detector (HP 1047 A, Hewlett-Packard, Palo Alto, CA, USA) and a UV/Vis detector (K-2600, Knauer). The device was controlled using EZ-Chrome Lite software (version 3.3.2, Agilent, Santa Clara, CA, USA). The sample injection volume was 20 μL, and the compounds were separated in a Rezex RoA-organic acid H^+^ (8%) resin-based column preceded by a SecurityGuard guard cartridge (Phenomenex). The separation of D-glucose, D-threose, glycolaldehyde, D-threonate/lactone and acetate was performed at 35 °C with 0.5 mM H_2_SO_4_ at 0.5 mL min^−1^ as mobile phase. For the measurement of DHB and ethylene glycol concentrations, the column was held at 80 °C under otherwise identical conditions. It is important to note that our HPLC method could not discriminate between D-threonate and D-threono-1,4-lactone due to the identical retention times of these metabolites. Since both compounds showed nearly identical peak area to molar concentration ratios, we make reference to the pooled concentration of D-threonate and D-threono-1,4-lactone in the shake flask cultures.

In reactor experiments, we used the hydroxamate method^55,56^ to resolve the two compounds. Specifically, 200 µL of cell-free supernatant were added to 400 µL of 2 M hydroxylamine solution (freshly prepared by dissolving hydroxylamine HCl in 2 M NaOH). After 2 min of incubation, 260 µL of 3.2 M HCl and subsequently 200 µL of FeCl_3_ solution (16 g FeCl_3_*6H_2_O in 100 mL of 0.1 M HCl) were added to generate ferric-hydroxamate complexes, which were detected by measuring corresponding optical densities at a wavelength of 550 nm. D-threono-1,4-lactone standards (Sigma-Aldrich) were used to prepare the calibration curve (see Supplementary Fig. 14). The specificity of the colorimetric assay was evaluated by testing standards of glycolaldehyde and major fermentation products threose, threonate, threono-1,4-lactone and DHB. The assay also responded to glycolaldehyde and threose, albeit with lower sensitivity that for threono-1,4-lactone (see Supplementary Fig. 14). Therefore, measurements of D-threonolactone concentrations were only carried out in samples not containing glycolaldehyde, and corrected for the presence of threose by subtracting OD_550_ values corresponding to the threose concentrations independently measured by HPLC. The start sample (*t* = 0 h) was used for subtracting the background absorbance caused by the medium. The concentration of D-threonate was calculated from the difference between the pooled concentration of D-threonate and D-threono-1,4-lactone and the measured lactone concentration.

Measurements of ^13^C enrichments in DHB were performed using a LC/MS platform, which consists of a Vanquish and a Thermo Scientific™ Q Exactive™ Focus (ThermoFisher Scientific), controlled by Xcalibur software (version 2.1, ThermoFisher Scientific). Separation by liquid chromatography was achieved using Rezex RoA-organic acid H^+^ (8%) resin-based column preceded by a SecurityGuard guard cartridge (Phenomenex) held at 80 °C with 0.1% formic acid as mobile phase. The temperature of the autosampler was kept at 6 °C, the injection volume was 20 µL and an isocratic flow of 0.4 mL min^−1^ was adjusted. Peak areas were corrected for the contribution of all naturally abundant isotopes using the software IsoCor^67^ (version 2.2.0).

### Analysis of isotopic labelling in intracellular metabolites

Intracellular metabolites were investigated with regard to their isotopomer distribution. Whenever appropriate, a volume of 1.5 mL of bacterial liquid culture was filtered through a polyamide 0.2 µm filter (Sartorius Stedim, Goettingen, Germany) and cells were washed with 3 mL room temperature water. The filter was then immediately transferred to a 10 mL glass tube containing 5 mL of hot ethanol (75% v/v) and vortexed for 10 s. Following 3 min of incubation at 80 °C, the glass vial was placed on ice, and the filter removed before the suspension was centrifuged (at 13,000 g for 5 min). The supernatant was stored overnight at -20 °C. Samples were dried at 40 °C for 4-5 h using a benchtop vacuum concentrator (CentriVap Concentrator System, Labconco, Kansas City, MO, USA). Dried metabolites were resuspended in 250 µL water and analyzed by LC/MS (Vanquish® and a Thermo Scientific™ Q Exactive™ Focus, ThermoFisher Scientific). Separation by liquid chromatography was achieved using a SeQuant® ZIC®-pHILIC (5 µm polymer 150 × 2.1 mm, Merck, Darmstadt, Germany) column with a flow rate of 0.15 mL min^-1^. For optimal separation, a gradient of A (5% ACN, 10 mM ammonium acetate, pH 9.2 by NH_4_OH) and B (90% ACN, 10 mM ammonium acetate, pH 9.2 by NH_4_OH) was used. The gradient was as follows: 0 min, 95% B; 2 min, 95% B; 3 min, 89.4% B; 5 min, 89.4% B; 6 min, 83.8% B; 7 min, 83.8% B; 8 min, 78.2% B; 9 min, 78.2% B; 10 min, 55.9% B; 12 min, 55.9% B; 13 min, 27.9% B; 16 min, 27.9% B; 18 min, 0% B; 23 min, 0% B; 24 min, 95% B; 30 min, 95% B. The temperature of the autosampler was kept at 6 °C, injection volume was 5 µL and oven temperature was kept at 25 °C. Instrumental settings according the electrospray ionization were optimized for a flow rate of 0.15 mL min^-1^. Final parameters were adjusted as follows: sheath gas flow rate 32 (arbitrary units), auxiliary gas flow rate 8 (arbitrary units), sweep gas flow rate 0 (arbitrary units), spray voltage -3.5 kV, capillary temperature 250 °C and auxiliary gas temperature 200 °C. With exception of 3-deoxy-alpha-D-manno-2-octulosonate (KDO), retention times of all shown analytes were verified by injecting unlabelled standards. Peak areas were corrected for the contribution of all naturally abundant isotopes using the software IsoCor^67^ (version 2.2.0).

### Reporting summary

Further information on research design is available in the Nature Portfolio Reporting Summary linked to this article.

## Supplementary information

Supplementary Information

Description of Additional Supplementary Files

Supplementary Data 1

Supplementary Data 2

Supplementary Data 3

Reporting Summary

## Data Availability

Data supporting the findings of this work are available within the paper and its Supplementary Information file. A reporting summary for this Article is available as a Supplementary Information file. Source data are provided with this paper.

## Source data

Source Data
