# Supplementary material for: Construction of a synthetic metabolic pathway for biosynthesis of 2,4-dihydroxybutyric acid from ethylene glycol
Source: Nat Commun. 2023 Apr 6;14:1931. doi: 10.1038/s41467-023-37558-x (PMC10079672; doi:10.1038/s41467-023-37558-x)
Supplement: Supplementary file 1 — Supplementary Information [file 41467_2023_37558_MOESM1_ESM.pdf]

Construction of a synthetic metabolic pathway for biosynthesis of 2,4-dihydroxybutyric acid from ethylene glycol

Frazão *et al.*

Supplementary Method 1. Standard Gibbs free energy of the DHB pathway

The stoichiometry of the reactions methanol dehydrogenase (MeD), glycolaldehyde synthase (GaLS), ethylene glycol dehydrogenase (EGD), D-threose aldolase (TA), D-threose dehydrogenase (TD), D-threonolactonase (TL), D-threonate dehydratase (TDH) and OHB reductase (OR) is as follows:

(MeD) methanol + NAD^+ = formaldehyde + NADH + H^+

(GaLS) 2 formaldehyde = glycolaldehyde

(EGD) ethylene glycol + NAD^+ = glycolaldehyde + NADH + H^+

(TA) 2 glycolaldehyde = (D)-threose

(TD) (D)-threose + $NAD(P)^+$ = (D)-threonolactone + NAD(P)H + H^+

(TL) (D)-threonolactone + H_2O = (D)-threonate$^-$ + H^+

(TDH) (D)-threonate$^-$ = OHB^- + H2O

(OR) OHB^- + NAD(P)H + H^+ = DHB^- + $NAD(P)^+$

The standard Gibbs free energy of a reaction ($\Delta_r G^{'0}$) was calculated from eQuilibrator (http://equilibrator.weizmann.ac.il/). For those reactions not available in eQuilibrator (TD, TL, TDH, OR), the standard Gibbs free energy for the formation of the DHB pathway intermediates ($\Delta_f G^{0'}$) was first calculated based on the group contribution theory using the dataset published by Jankowski *et al.* [1] (Supplementary Table 1). The standard Gibbs free energy of a reaction ($\Delta_r G^{0'}$) was then estimated according to Equation (**1**) from the standard Gibbs free energy of the formation of the participating compounds ($\Delta_f G^{0'}$), with v_i being the stoichiometric coefficients. All values were estimated assuming pH 7 and metabolite concentrations at 1M.

$$\Delta_r G^{0\prime} = \Sigma_i v_i \, . \, \Delta_f G_i^{0\prime} \qquad \textbf{(1)}$$

Accordingly, the standard Gibbs free energy of the investigated reactions is as follows, MeD: 33.6 kJ mol^{-1}; GaLS: -45.8 kJ mol^{-1}; EGD: 23.7 kJ mol^{-1}; TA: -16.1 kJ mol^{-1}; TD: -24.8 kJ mol^{-1}; TL: -15.1 kJ mol^{-1}; TDH, -44.5 kJ mol^{-1}; OR, -15.7 kJ mol^{-1}.

The pathway starting from glycolaldehyde, ethylene glycol or methanol has a negative standard Gibbs free energy (respectively -116.2 kJ mol^{-1}, -92.5 kJ mol^{-1} or -128.4 kJ mol^{-1}).

Supplementary Method 2. Construction of a cytoplasmic lactonase variant derived from Tt.Lac11

The gluconolactonase from *Thermogutta terrifontis* (UniprotKB, A0A286RDQ9; Tt.Thte1497, herein denoted Tt.Lac11) has previously been shown to display promiscuous activity towards a wide range of substrates, including D-glucono-1,5-lactone, D-glucuronic acid-1,4-lactone, D-threono-1,4-lactone or L-fucono-1,4-lactone[2]. Tt.Lac11 protein has previously been expressed in *E. coli* BL21-CodonPlus (DE3) RIPL cells, and purified from the soluble fraction, which contains both periplasmic and cytosolic proteins. Taking into account the presence of multiple rare codons in respective natural DNA sequence as assessed with GCUA online tool (https://gcua.schoedl.de), we ordered a codon-optimized synthetic gene which was cloned into appropriate cloning sites of pET28a vector to yield His_6-tagged protein at N- or C- terminus. At variation with Westlake[2], we obtained low concentrations of purified protein ($\leq$ 0.2 mg ml^{-1}) from *E. coli* BL21(DE3) modified cells even though *in vitro* activity of purified enzyme with D-threono-1,4-lactone could indeed be confirmed (Supplementary Table 7) and kinetic parameters estimated (Table 2). When we further attempted DHB biosynthesis from glycolaldehyde (Table 5) or D-threose (Supplementary Figure 8) with *E. coli* MG1655-derived producer strains, we observed (increased) product formation (0.08 mM from 20 mM glycolaldehyde; 4.76 mM from 10 mM D-threose) strictly dependent on the expression D-threonate importer Re.KdgT. In absence of heterologous expression of Re.KdgT, we observed extracellular accumulation of D-threonate/lactone regardless of Tt.Lac11 expression thereby suggesting lactone hydrolysis to mostly likely occur outside the cytosol. We further carried out bioinformatic analyses to ascertain the cellular location of Tt.Lac11 (Supplementary Figure 7). In particular, a signal peptide sequence is predicted to occur at the N-terminus of the protein sequence with cleavage site between positions 37-38 (Phobius[3], TargetP-2.0[4]) (Supplementary Figures 7a, 7c). While signal peptide presence is typical in periplasmic proteins, we did not find a particular good fit between that of Tt.Lac11 and consensus sequences of prokaryotic Sec- or TAT-signal peptides (Supplementary Figure 7c). Finally, we used BlastP to identify protein domains in Tt.Lac11 having found domains belonging to the sugar lactone lactonase YvrE (COG3386; positions 52-355) and SMP-30/Gluconolactonase/LRE-like family (SGL) (Pfam_08450; residues 77-355) family of enzymes (Supplementary Figure 7b).

Taking into account that a cytoplasmic lactonase would avoid DHB pathway intermediates export and subsequent re-import, we therefore set out to create a cytoplasmic version of Tt.Lac11 by deleting variable segments of corresponding N-terminus sequence while adding a start ATG codon to each sequence. We

constructed three variants (v1-v3) of the protein fused *in-frame* to His_6-tag at C-terminus that were cloned in pET28a vectors, in which Tt-Lac11^{v1} is deleted for signal peptide sequence (Δ1-38 aa), Tt.lac11^{v2} contains YrvE domain only (Δ1-51 aa) and Tt.Lac11^{v3} harbours SGL domain only (Δ1-77 aa). After protein expression and purification from *E. coli* BL21(DE3) cells, we found Tt.Lac11^{v1} and Tt.Lac11^{v2} to be expressed at higher levels than wild-type enzyme, as well as strongly improved *in vitro* activities on synthetic substrate (Supplementary Table 7). Taking into account that Tt.Lac11^{v1} was the variant with the highest activity on D-threono-1,4-lactone (12.35 U mg^{-1}) among all tested candidates, we retained the enzyme for further experiments. Next, we expressed the new enzyme variant in DHB-producing cells devoid of D-threonate importer having found strongly improved product formation when using glycolaldehyde or D-threose as pathway substrates (Table 5, Supplementary Figure 8). Lastly, we confirmed Tt.Lac11^{v1} as a cytosolic protein by separating and analysing subcellular fractions of *E. coli* cells expressed the corresponding gene (Supplementary Figure 9).

Supplementary Method 3. Subcellular fractionation of lactonase-expressing BL21(DE3) strains.

Periplasmic fraction

After protein expression in a volume of 50 mL as described in Supplementary Table 11, cells were harvested by centrifugation (20 min at 8,000 g, 4 ºC) and supernatant was discarded. Extraction of periplasmic proteins was carried out immediately after and was based on protocols available elsewhere[5,6]. Briefly, the cell pellet was carefully resuspended in TSE buffer (200 mM Tris-HCl, pH 8, 500 mM sucrose, 1 mM EDTA) that was added at 12 µL per OD_{600} unit. The suspension was incubated for 10 min at room temperature, after which cells were cold-shocked by addition of ice-cold water (added at 12 µL per OD_{600} unit). The mixture-containing tube was immediately transferred to ice for an additional period of 10 min, and followed by centrifugation (20 min at 8,000 g, 4 ºC). The supernatant which contains periplasmic fraction was carefully collected.

Cytosolic, soluble fraction

The remaining cell pellet was resuspended in 1 mL of lysis buffer (50 mM Hepes, pH 7.5, 300 mM NaCl) and broken open by four successive rounds of sonication (TOPAS, Ultrasonic Disintegrator UDS 751) with the power output set to 40 %. Cell debris was removed by centrifuging the suspension (15 min at 15,493 g, 4 ºC) and retaining the clear supernatant containing cytosolic fraction.

Insoluble fraction

The remaining pellet was resuspended in 1 mL of solubilisation buffer (50 mM Hepes, pH 7.5, 300 mM NaCl, sarcosyl 10 %), followed by centrifugation for 10 min at 11,300 g. The supernatant was kept for posterior analysis of insoluble proteins.

Supplementary Method 4. Enzyme assays

EG dehydrogenase

The enzyme activity was assayed in the oxidative direction by monitoring reduction of NAD^+ at 340 nm (ε = 6.22 mM^{-1} cm^{-1}) during oxidation of ethylene glycol. The experiment was based on the procedure originally described by Boronat *et al.* [7]. The assay mixture contained 100 mM sodium glycine (pH 9.5), 0.5 mM NAD^+, and appropriate amounts of crude protein extract. Reactions were started by adding 50 mM of substrate. One unit of EG dehydrogenase activity (U) was defined as the amount of enzyme catalyzing the conversion of 1.0 µmole of NAD^+ per minute.

Sugar dehydrogenase

the enzyme activity was assayed in the oxidative direction by monitoring reduction of $NAD(P)^+$ at 340 nm during oxidation of candidate sugars in an experiment adapted from the procedure described by Hobbs and colleagues (2014)[8]. The assay mixture contained 50 mM Hepes (pH 8), 10 mM $NAD(P)^+$, and appropriate amounts of purified enzyme. Reactions were started by adding variable concentrations of (D)-arabinose or (D)-threose (Carbosynth, UK). One unit of sugar dehydrogenase activity (U) was defined as the amount of enzyme catalyzing the conversion of 1.0 µmole of $NAD(P)^+$ per minute.

Lactonase

The enzyme activity was assayed by monitoring protons released from the carboxylate product during hydrolysis of lactones using the colorimetric pH indicator bromothymol blue whose absorbance was read at 616 nm (ε = 1.14 mM^{-1} cm^{-1}). The experiment was adapted from a procedure described by Hobbs and colleagues (2014)[8]. The assay mixture contained 2.5 mM Hepes (pH 7.1), 200 mM NaCl, 1% (v/v) DMSO, 0.1 mM bromothymol blue and appropriate amounts of purified enzyme. The reaction was started by addition of variable amounts of (L)-fucono-1,4-lactone or (D)-threono-1,4-lactone. One unit of lactonase activity (U) was defined as the amount of enzyme catalyzing the hydrolysis of 1.0 µmole of lactone per minute.

Sugar acid dehydratase

In our dehydratase screening adapted from Tai *et al.*[9], enzyme activity was assayed by converting the 2-keto acid reaction product to a semicarbazone which could be detected at 250 nm. The assay was calibrated using the 2-keto acid pyruvate as external standard (ε = 2.24 mM^{-1} cm^{-1}). The assay mixture contained 60 mM Hepes (pH 7.3), 50 mM KCl, 10 mM $MgCl_2$ and appropriate amounts of purified enzyme. The reactions were performed at 37 °C in volumes of 1 mL and were started by addition of sugar acid (L-fuconate, 2R-dihydroxivalerate, D-altronate, D-tartrate, D-arabinonate or D-threonate). Aliquots of 200 µL were taken at 0, 10, 20 and 40 minutes and quenched with 100 µL of 2 M HCl. Samples were then supplemented with 300 µL of semicarbazide solution (10 g L^{-1} semicarbazide hydrochloride and 15 g L^{-1} sodium acetate) and incubated at 30 °C for 10 minutes. Finally, 500 µL of distilled water were added to the derivatized product, and absorbance was immediately measured using a quartz cuvette. One unit of sugar acid dehydratase activity (U) was defined as the amount of enzyme catalyzing the formation of 1.0 µmole of 2-keto acid per minute.

D-Threonate dehydratase

The enzyme activity was assayed by coupling dehydration of D-threonate to NADH-dependent reduction of the reaction product 2-keto-4-hydroxybutyrate (OHB). The previously described L-malate dehydrogenase mutant Ec.Mdh^{5Q} was used as OHB reductase[10]. The reaction mixture contained 60 mM Hepes (pH 7.3), 50 mM KCl, 10 mM $MgCl_2$, 0.25 mM NADH, 100 µg mL^{-1} of auxiliary enzyme and appropriate amounts of purified enzyme. The reaction was started by the addition of variable amounts of substrate. One unit of D-threonate dehydratase activity (U) was defined as the amount of enzyme catalyzing the formation of 1.0 µmole of OHB per minute.

Supplementary Method 5. Plasmid construction

Ethylene glycol to glycolaldehyde module

Plasmids pEXT20-Ec.fucO, pEXT20-Ec.$fucO^{I6L:L7V}$ and pEXT20-Go.adh were constructed by PCR-amplifying the Ec.*fucO* wild-type, Ec.$fucO_{I6L:L7V}$ and codon-optimized Go.*adh* genes using, respectively, primer pairs 224/223, 222/223 and 315/226. Genomic DNA of *E. coli* MG1655 and a synthetic gene served as template, respectively, for Ec.*fucO* derived genes and Go.*adh*. All primers introduced unique restriction sites flanking the gene of interest, and additionally forward primer inserted a ribosome binding sequence (RBS) immediately upstream coding sequence. The high-copy number pEXT20 expression vector[11], which served as backbone, was

amplified using primer pair 209/284. PCR products were digested with XhoI / XbaI restriction enzymes and ligated to digested vector backbone using T4 DNA ligase (New England Biolabs).

Glycolaldehyde to D-threonate module

Plasmids pEXT20-Ec.fsaA and pEXT20-Ec.fsaA^{TA} were constructed by amplifying the Ec.*fsaA* wild-type and Ec.$fsaA_{L107Y:A129G}$ genes using the primer pair 326/327. Genomic DNA of *E. coli* MG1655 and synthetic gene served, respectively, as template. The resulting PCR products and pEXT20 expression vector were digested with BamHI / XbaI and ligated. Plasmid pEXT20-Ec.fsaA^{TA}-Pc.tadH was constructed by PCR-amplifying Ec-$fsaA_{L107Y:A129G}$ and codon-optimized Pc.*tadH* synthetic genes using the primer pairs 326/328 and 303/304, respectively. Resulting PCR products were digested with BamHI / SwaI and SwaI / Xbai, respectively, and ligated to BamHI / XbaI digested pEXT20 vector. A similar procedure was followed for construction of pACT3-Ec.fsaA^{TA}-Pc.tadH, in which the medium-copy number pACT3 plasmid served, however, as backbone. Plasmid pACT3-Ec.fsaA^{TA}-Pc.tadH-Tt.lac11 was constructed by amplifying the codon-optimized Tt.*lac11* synthetic gene using the primer pair 438/439. Both the PCR product and pACT3-Ec.fsaA^{TA}-Pc.tadH vector were then digested with XbaI / SalI and ligated. A shorter version of Tt.*lac11* gene missing protein-export signal peptide sequence at the N-terminus (Δ115-1,068 nt), named as Tt.*lac11*^{v1}, was likewise PCR-amplified using primer pair 600/439. Ligation of XbaI / SalI digested PCR product and pACT3-Ec.fsaA^{TA}-Pc.tadH yielded plasmid pACT3-Ec.fsaA^{TA}-Pc.tadH-Tt.lac11^{v1}.

D-threose to D-threonate module

Plasmid pACT3-Pc.tadH was constructed by amplifying codon-optimized Pc.*tadH* gene using primer pair 874/304. Ligation of BamHI / XbaI digested PCR product and pACT3 plasmid yielded desired construct. Plasmids pACT3-Pc.tadH-Tt.lac11 and pACT3-Pc.tadH-Tt.lac11^{v1} were constructed by first amplifying by PCR variable parts of Tt.*lac11* gene using primer pairs 438/439 and 600/439, respectively. DpnI-treated PCR products, and pACT3-Pc.tadH were digested with XbaI / SalI restriction enzymes and ligated.

Ethylene glycol to D-threonate module

Plasmid pACT3-Go.adh-Ec.fsaA^{TA}-Pc.tadH-Tt.lac11^{v1} was constructed by amplifying the Go.*adh*, Ec.*fsaA*^{TA}, Pc.*tadH* and Tt.*lac11*^{v1} genes using, respectively, primer pairs 313/314, 326/328, 303/304 and 600/439.

Resulting PCR products were digested with KpnI / BamHI, BamHI / SwaI, SwaI / XbaI and XbaI / SalI, respectively, and ligated to KpnI / SalI digested pACT3 vector.

D-threonate to DHB module

Plasmid pEXT22-Ec.mdh^{5Q} was constructed by PCR-amplifying the OHB reductase[10] encoding gene Ec.*mdh*$_{5Q}$ synthetic gene using primer pair 305/258. The resulting PCR product and the low-copy number pEXT22 vector were then digested with SacI / BamHI and ligated. Plasmids pEXT22-Ec.mdh^{5Q}-Aa.araD and pext22-Ec.mdh^{5Q}-Hh.araD were constructed by amplifying Aa.*araD* and Hh.*araD* genes using primer pairs 551/552 and 553/554, respectively. Genomic DNAs of *Acidovorax avenae* DSM7227 and *Herbaspirillum huttiense* DSM10281 were used as the respective templates. The resulting PCR products were digested with BamHI / XbaI and individually ligated to corresponding sites in BamHI/XbaI digested vector pEXT22-Ec.mdh^{5Q}.

D-threonate importer module

Plasmid pEXT21-Re.kdgT was constructed by amplifying Re.*kdgT* gene from genomic DNA of *Cupriavidus necator* H16 DSM428 using primer pair 454/455. PCR product and pEXT21 vector backbone were digested with BamHI / HindIII restriction enzymes and ligated.

D-threonate assimilation pathway

Plasmid pEXT22-Re.dtnK-Re.pdxA2 was constructed by PCR-amplifying the Re.*dtnK* and Re.*pdxA2* genes using primer pairs 397/398 and 399/400, respectively. Genomic DNA of *Ralstonia eutropha* H16 (DSM428) served as template. The resulting PCR products were digested with EcoRI / XbaI (Re.*dtnK*) or XbaI / BamHI (Re.*pdxA2*) and ligated to EcoRI / BamHI digested pEXT22 expression vector backbone using T4 DNA ligase (New England Biolabs). Plasmid pEXT22-Re.dtnK-Re.pdxA2-Re.kdgT was constructed by amplifying Re.*kdgT* gene from genomic DNA of *C. necator* H16 (DSM428) using primer pair 454/455. PCR product and pEXT22-Re.dtnK-Re.pdxA2 plasmid were digested with BamHI / HindIII restriction enzymes and ligated.

Supplementary Table 1. Gibbs free energy of formation of DHB pathway intermediates. Values were estimated manually according to Jankowski *et al*.[1].

**Compound**	**Contributing groups**	**$\Delta_f G^{0'}$ [kJ mol^{-1}]**
(D)-threose	1x –CH=O<br>3x -OH<br>2x >CH-<br>1x >CH_2-	-600.0
(D)-threonolactone	1x >CH2 (in one ring)<br>1x –O- (in a ring)<br>3x -OH<br>2x >CH- (in a ring)<br>1x >CH_2 (in a ring)	-606.9
(D)-threonate$^-$	1x -COO1$^-$<br>3x -OH<br>2x >CH-<br>1x –CH_2-	-819.3
OHB$^-$	1x -COO^{1-}<br>1x –OH<br>2x -CH_2-<br>1x >CO	-626.6
DHB$^-$	1x -COO^{1-}<br>2x –OH<br>2x -CH_2-<br>1x –CH<	-660.2
H^+		-39.9
NADH		-2193.8
NAD^+		-2215.8
H_2O		-237.2

Supplementary Table 2. Specific activities (expressed in U mg of purified enzyme^{-1}) of N-his tagged candidate D-threose dehydrogenase enzymes. Activities were measured at a pH of 8 with fixed amount (10 mM) of substrate (D-arabinose or D-threose) and co-factor (NAD^+ or $NADP^+$). Results are expressed as mean (±STDV) of two biological replicates. Legend: n.d., not detected; n.a., information not available or found.

**Enzyme**	**Optimum pH**	**D-arabinose (U mg^{-1})**		**D-threose (U mg^{-1})**	
		**NAD^+**	**$NADP^+$**	**NAD^+**	**$NADP^+$**
Sc.Ara1	10.0 [a]	0.02 (±0.006)	0.52 (±0.18)	n.d.	0.02 (±0.007)
Sc.Ara2	8.2 [b]	n.d.	0.12 (±0.05)	n.d.	n.d.
Pl.LgdA	n.a. [c]	n.d.	n.d.	n.d.	n.d.
Pc.TadH	10.0 [d]	4.65 (±0.20)	0.86 (±0.22)	0.75 (±0.02)	n.d.
Bm.Fdh	n.a. [e]	0.05 (±0.01)	n.d.	0.06 (±0.005)	n.d.
Ps.Fdh	9.0-10.5 [f]	n.d.	0.82 (±0.28)	n.d.	n.d.
Ss.Adh4	8.2 [g]	n.d.	0.053 (±0.075)	n.d.	n.d.

[a] Enzyme activity was tested in a pH range of 4-10 by van Bergen *et al.* [12], with the highest dehydrogenase activity being found at pH 10.0.

[b] See work of Amako *et al.*[13].

[c] Enzyme activity has previously been only tested at pH 8 by Shimizu *et al.*[14].

[d] See work of Sasajima *et al.*[15].

[e] Enzyme activity has previously only been tested at pH 8 by Hobbs *et al.*[8].

[f] See work of Horiuchi *et al.*[16].

[g] See work of Brouns *et al* .[17].

Supplementary Table 3. Kinetic parameters of N-his tagged candidate D-threose dehydrogenase Pc.TadH on natural and synthetic substrates. The gene encoding the referred enzyme was codon optimized by Eurofins SAS. Results are expressed as mean (±STDV) of two biological replicates.

Enzyme	D-arabinose		D-threose	
	NAD^+	$NADP^+$	NAD^+	$NADP^+$
V_{max} (U mg^{-1})	2.77 (±0.34)	1.60 (±0.45)	3.50 (±0.10)	-
K_m (mM)	0.23 (±0.05)	17.20 (±3.40)	-	-
V_{max}/K_m (U mg^{-1} M^{-1})	12,043	93	70	-

Supplementary Table 4. Kinetic parameters of N-his tagged candidate D-threonolactonase Tt.Lac11 on natural and synthetic substrates. The gene encoding the referred enzyme was codon optimized by Eurofins SAS. Results are expressed as mean (±STDV) of two biological replicates.

Substrate	Kinetic parameters		
	V_{max} (U mg^{-1})	K_m (10^{-3} M)	V_{max}/K_m (U mg^{-1} M^{-1})
L-fucono-1,4-lactone	1.18 (±0.04)	0.84 (±0.06)	1,405
D-threono-1,4-lactone	1.76 (±0.16)	2.92 (±0.13)	603

Supplementary Table 5. Specific activities (expressed in U mg of purified enzyme^{-1}) of N-his tagged candidate D-threonate dehydratase enzymes. Activities were measured with fixed amount (10 mM) of natural or synthetic D-threonate substrate. The assay mixture additionally contained 60 mM Hepes (pH 7.3), 50 mM KCl and 10 mM $MgCl_2$. Results are expressed as the mean (±STDV) of two biological replicates. Legend: n.d., not detected.

Enzyme	Natural substrate	Activity on natural substrate (U mg^{-1})	Activity on D-threonate (U mg^{-1})
Ec.IlvD	2R-dihydroxyvalerate 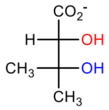	0.88 (±0.32)	n.d.
Ss.IlvD	2R-dihydroxyvalerate 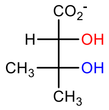	0.01 (±0.004)	n.d.
Xc.FucD	L-fuconate 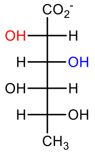	4.03 (±1.07)	n.d.
Pp.FucD	L-fuconate 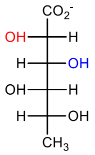	n.d.	n.d.
Bj.TarD	D-tartrate 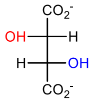	4.68 (±0.67)	n.d.
Aa.AraD	D-arabinonate 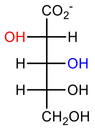	0.30 (±0.06)[a]	0.18 (±0.08)
Hh.AraD	D-arabinonate 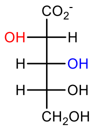	0.58 (±0.04)[a]	0.30 (±0.02)
Ec.UxaA	D-altronate 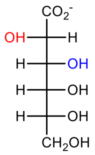	0.07 (±0.05)[a, b]	n.d.

[a] Enzyme activity tested with substrate at a final concentration of 1 mM.

[b] Enzyme activity estimated in the presence of $FeSO_4$ (0.5 mM). No activity was observed in the presence of $MgCl_2$ (10 mM) or $MnSO_4$ (10 mM).

Supplementary Table 6. Kinetic parameters of N-his tagged D-threonate dehydratases on multiple substrates, including D-threonate. Results from this study are expressed as the mean (±STDV) of two biological replicates. Legend: MW, molecular weight.

Enzyme	Substrate	V_{max} (U mg^{-1})	k_{cat} (min^{-1})	K_m (mM)	V_{max}/K_m (U mg^{-1} M^{-1})	k_{cat} / K_m (min^{-1} mM^{-1})	Source
Hh.AraD (MW: 54.47 kDa)	D-threonate	0.19 (±0.04)	10.3 (±2.2)	1.21 (±0.36)	157	8	This work
	D-arabinonate	-	563	1.35	-	419	[18]
	L-xylonate	-	360	1.02	-	354	
	D-altronate	-	55.7	1.27	-	44	
	D-idonate	-	16.1	0.29	-	56	
	L-fuconate	-	12.1	0.88	-	14	
	L-gluconate	-	29.9	0.99	-	31	
Aa.AraD (MW: 52.7 kDa)	D-threonate	0.12 (±0.06)	6.3 (±3.2)	1.95 (±0.53)	62	3	This work
	D-arabinonate	-	194	0.31	-	631	[18]
	L-xylonate	-	40.2	0.51	-	80	
	D-altronate	-	21.1	0.31	-	68	
	L-fuconate	-	4.9	0.12	-	43	
	D-idonate	-	4.2	0.23	-	19	
	L-gluconate	-	4.3	0.12	-	37	

Supplementary Table 7. Protein expression of his-tagged versions of Tt.Lac11 from pET28a-derived vector in *E. coli* BL21(DE3) cells. The gene was codon-optimized and expressed with N- or C-terminal his-tag as indicated below. Results are expressed as the mean (±STDV) of two biological replicates. Legend: n.d., not detected.

Variant	His-tag	Protein expressed	Conc. [a] (mg mL^{-1})	Activity (U mg^{-1}) [b] on 4 mM substrate	
				L-Fucono-1,4-lactone	D-threono-1,4-lactone
Tt.Lac11	N-term	wt	0.142	1.21 (±0.22)	1.49 (±0.23)
Tt.Lac11	C-term	wt	0.211	4.22 (±0.28)	4.71 (±1.83)
Tt.Lac11^{v1}	C-term	Δ1-38	4.886	16.46 (±2.01)	12.35 (±1.97)
Tt.Lac11^{v2}	C-term	Δ1-51	2.076	12.10 (±0.85)	7.65 (±0.04)
Tt.Lac11^{v3}	C-term	Δ1-76	0.134	n.d.	0.21

[a] Concentration expressed as mg purified protein mL^{-1}.

[b] Activity expressed as U mg purified protein^{-1}.

Supplementary Table 8. Ethylene glycol dehydrogenase activities[a] of candidate genes expressed in *E. coli* MG1655 Δ*aldA* Δ*yqhD* host strain. Data shown refer to one single experiment. Legend: n.d., not detected.

Strain	Plasmid	Activity (µmol min^{-1} mg^{-1}) [b]
TW138	pEXT20	n.d.
TW139	pEXT20-Ec.fucO	0.162
TW140	pEXT20-Ec.fucO$^{I6L:L7V}$	0.203
TW141	pEXT20-Go.adh	0.076

[a] One unit of activity equals 1 µmol of NADH formed per min at 37 °C. Cells were cultivated in M9 mineral medium containing 20 g L^{-1} of glucose and supplemented with 10% (v/v) LB (starting OD, 0.2). IPTG (0.5 mM) was added when OD ~0.6. Cells (40 OD units) were harvested when an optical density at 600 nm of 2.0 was reached.

[b] Activities were expressed in µmol per min per mg of clarified cell extract, and determined in the presence of substrate at 50 mM final concentration in a reaction mixture containing sodium glycine (100 mM, pH 9.5) and NAD^+, 0.5 mM.

Supplementary Table 9. Summary of EG-to-DHB bioconversion experiments. Results are shown for total incubation periods of 48 h. Legend: n.d. - not detected; n.m. - not measured.

Condition	Strain	EG added	EG consumed [mM]	GA [mM]	Threose [mM]	Threonate /lactone [mM]	DHB [mM]	DHB yield [mol mol^{-1}]	C-balance (%)
**Growing cells** [a]									
M9 (90% v/v) + LB (10% v/v)	TW363	0 mM	-	n.d.	0.06 (±0.001)	n.d.	n.d.	-	-
		160 mM	6.37 (±1.18)	n.d.	0.03 (±0.03)	n.d.	n.d.	-	-
		320 mM	9.61 (±1.00)	0.40 (±0.03)	0.11 (±0.08)	n.d.	0.41 (±0.07)	0.05	15
		640 mM	14.0 (±4.27)	3.87 (±0.0003)	0.37 (±0.11)	n.d.	0.29 (±0.14)	0.02	37
M9 (90% v/v) + LB (10% v/v)	TW1828	320 mM	8.70 (±0.46)	n.d.	0.57 (±0.02)	0.30 (±0.02)	0.80 (±0.06)	0.09	38
LB	TW363	320 mM	n.m.	n.m.	n.m.	n.m.	0.74	-	-
LB (glucose, 4 g/l)	TW363	320 mM	n.m.	n.m.	n.m.	n.m.	0.45	-	-
LB (glucose, 20 g/l)	TW363	320 mM	n.m.	n.m.	n.m.	n.m.	0.41	-	-
**Resting cells** [b]									
M9 (no added co-substrate)	TW1828	320 mM	40.06 (±3.86)	1.56 (±0.02)	3.41 (±0.65)	15.18 (±0.12)	2.14 (±0.34)	0.05	107
M9 (glucose beads)	TW1828	320 mM	36.40 (±2.53)	n.d.	1.31 (±0.02)	13.74 (±0.10)	3.16 (±0.0006)	0.08	92
M9 (glucose beads) + L-cysteine + $FeCl_3$	TW1828	320 mM	41.47 (±8.01)	n.d.	1.89 (±0.04)	14.18 (±0.45)	2.82 (±0.04)	0.07	91
LB	TW1828	320 mM	46.03 (±0.56)	n.d.	1.58 (±0.17)	7.84 (±0.96)	5.66 (±0.81)	0.12	66
LB + L-cysteine + $FeCl_3$	TW1828	320 mM	45.37 (±0.40)	n.d.	1.66 (±0.09)	6.18 (±0.01)	6.75 (±0.16)	0.15	64

[a] Cells were grown at 37 °C, 220 rpm in 25 mL indicated medium with a starting OD_{600} = 0.2. IPTG (0.5 mM) and EG (at indicated amounts) were added when OD_{600} reached ~0.6. Results are expressed as mean (±STDV) of two biological replicates.

[b] Cells were grown at 37 °C, 220 rpm in 200 mL mineral medium supplemented with LB at 10% (v/v) with a starting OD_{600} = 0.2. IPTG (0.5 mM) was added when OD_{600} reached ~0.6. After 6.5 h, cells were concentrated to OD_{600} = 15 in 5 mL of indicated medium containing EG (320 mM) and IPTG (0.5 mM) and incubated at 37 °C, 220 rpm. In conditions "M9 (glucose beads)", addition of one glucose feed-bead was carried to facilitate the controlled release of the sugar (with a feed rate of ~0.5 mM or 0.09 g L^{-1} per hour, corresponding to a total added concentration of 25 mM or 4.5 g L^{-1}). Where indicated, medium was further supplemented with L-cysteine hydrochloride (1 mM) and $FeCl_3$ (0.06 mM).

Supplementary Table 10. Candidate enzymes selected for screening of missing activities of the synthetic DHB pathway.

Enzyme	Source	Function annotation	UniProt KB	Codon opt.
**Candidate D-threose dehydrogenases**				
Sc.Ara1	*S. cerevisiae*	NADP-dependent D-arabinose dehydrogenase	P38115	No
Sc.Ara2	*S. cerevisiae*	NAD-dependent D-arabinose 1-dehydrogenase	Q04212	No
Pc.TadH	*P. caryophylli*	D-threo-aldose 1-dehydrogenase	A0A1X7DDC2	Yes
Pl.LgdA	*P. laeviglucosivorans*	Scyllo-inositol dehydrogenase	K7ZP76	Yes
Ps.Fdh	*Pseudomonas* sp. 1143	D-threo-aldose 1-dehydrogenase	Q52472	Yes
Ss.Adh4	*S. solfataricus*	D-arabinose dehydrogenase	Q97YM2	Yes
Bm.Fdh	*B. multivorans*	NADP-dependent L-fucose dehydrogenase	A0A0H3KNE7	No
**Candidate D-threono-1,4-lactonase**				
Tt.Lac11	*T. terrifontis*	Gluconolactonase	A0A286RDQ9	Yes
**Candidate D-threonate dehydratases**				
Ec.IlvD	*E. coli*	Dihydroxy-acid dehydratase	P05791	No
Ss.IlvD	*S. solfataricus*	Dihydroxy-acid dehydratase	Q97UB2	Yes
Xc.FucD	*X. campestris*	L-fuconate dehydratase	Q8P3K2	Yes
Pp.FucD	*P. putida*	L-fuconate dehydratase	Q88J18	No
Bj.TarD	*B. japonicum*	D-tartrate dehydratase	Q89FH0	No
Aa.AraD	*A. avenae*	D-arabinonate dehydratase	F0Q4R8	No
Hh.AraD	*H. huttiense*	D-arabinonate dehydratase	A0A4P7ADP2	No
Ec.UxaA	*E. coli*	D-altronate dehydratase	P42604	No

Supplementary Table 11. Conditions used for expression of his-tagged proteins from pET28a-derived vector in *E. coli* BL21(DE3) cells. Following an overnight culture in 10 mL lysogeny broth (LB) medium, protein expression was evaluated in three conditions. Only those yielding the highest expression levels are shown below. Protein purity was visually inspected by running SDS-PAGE gels.

**Protein**	**Best condition for expression**
Ec.Mdh^{5Q}	50 mL LB with OD_i = 0.05.<br>IPTG inducer (1 mM) was added at OD ~0.6 and protein was expressed at 37 °C for 3h
Tt.Lac11 (N-tag)<br>Tt.Lac11 (C-tag)<br>Tt.Lac11^{v1} (C-tag)<br>Tt.Lac11^{v2} (C-tag)<br>Tt.Lac11^{v3} (C-tag)	50 mL Auto-induction medium[a] with OD_i = 0.05.<br>Protein was expressed at 25 °C for 24h
Sc.Ara1<br>Sc.Ara2<br>Pc.TadH<br>Pl.LgdA<br>Ps.Fdh<br>Ss.Adh4<br>Bm.Fdh	50 mL Auto-induction medium[a] with OD_i = 0.05.<br>Protein was expressed at 25 °C for 24h
Ec.IlvD	50 mL LB with OD_i = 0.05.<br>IPTG inducer (1 mM) was added at OD ~0.6 and protein was expressed at 37 °C for 3h
Ss.IlvD<br>Xc.FucD	50 mL LB with OD_i = 0.05.<br>IPTG inducer (0.5 mM) was added at OD ~0.6 and protein was expressed at 16 °C for 16h
Pp.FucD	No protein under any tested conditions
Bj.TarD<br>Aa.AraD<br>Hh.AraD	50 mL Auto-induction medium[a] with OD_i = 0.05.<br>Protein was expressed at 25 °C for 24h
Ec.UxaA	50 mL LB with OD_i = 0.05.<br>IPTG inducer (1 mM) was added at OD ~0.6 and protein was expressed at 37 °C for 3h

[a] Auto-induction medium was prepared as in https://openwetware.org/wiki/Lidstrom:Autoinduction_Media

Supplementary Table 12. Plasmids for bioproduction efforts used in this study. Sources of employed genes are abbreviated as follows: Ec, *Escherichia coli*; Go, *Gluconobacter oxydans*; Pc, *Paraburkholderia caryophylli*; Tt, *Thermogutta terrifontis*; Hh, *Herbaspirillum huttiense;* Aa, *Acidovorax avenae*; Re, *Ralstonia eutropha*.

**Plasmids**	**Relevant characteristics**	**Source**
pET28a(+)	Kan^R, f1 ori ; IPTG-inducible promoter T7	Novagen™
pEXT21	$Spec^R$, IncW ori; IPTG-inducible promoter TAC	[11]
pEXT22	Kan^R, IncFII ori; IPTG-inducible promoter TAC	[11]
pACT3	Chm^R; p15A ori; IPTG-inducible promoter TAC	[11]
pEXT20	Amp^R; colE1 ori; IPTG-inducible promoter TAC	[11]
pEXT20-Ec.fucO	pEXT20 derivative carrying Ec.*fucO*	This work
pEXT20-Ec.fucO$^{I6L:L7V}$	pEXT20 derivative carrying Ec.*fucO*$_{I6L:7LV}$	This work
pEXT20-Go.adh	pEXT20 derivative carrying Go.*adh* (= Go.*gox0313*, codon optimized)	This work
pEXT20-Ec.fsaA^{TA}	pEXT20 derivative carrying Ec.*fsaA*$_{L107Y:A129G}$	This work
pEXT20-Ec.fsaA^{TA}-Pc.tadH	pEXT20 derivative carrying Ec.*fsaA*$_{L107Y:A129G}$, Pc.*tadH* (codon optimized)	This work
pACT3-Ec.fsaA^{TA}-Pc.tadH	pACT3 derivative carrying Ec.*fsaA*$_{L107Y:A129G}$, Pc.*tadH* (codon optimized)	This work
pACT3-Ec.fsaA^{TA}-Pc.tadH-Tt.lac11	pACT3 derivative carrying Ec.*fsaA*$_{L107Y:A129G}$, Pc.*tadH* (codon optimized); Tt-*lac11* (codon optimized)	This work
pACT3-Ec.fsaA^{TA}-Pc.tadH-Tt.lac11^{v1}	pACT3 derivative carrying Ec.*fsaA*$_{L107Y:A129G}$, Pc.*tadH* (codon optimized); Tt-*lac11* (codon optimized; Δ1-38 aa)	This work
pACT3-Go.adh-Ec.fsaA^{TA}-Pc.tadH-Tt.lac11^{v1}	pACT3 derivative carrying Go.*adh* (= Go.*gox0313*, codon optimized), Ec.*fsaA*$_{L107Y:A129G}$, Pc.*tadH* (codon optimized); Tt-*lac11* (codon optimized; Δ1-38 aa)	This work
pACT3-Pc.tadH	pACT3 derivative carrying Pc.*tadH* (codon optimized)	This work
pACT3-Pc.tadH-Tt.lac11	pACT3 derivative carrying Pc.*tadH* (codon optimized); Tt-*lac11* (codon optimized)	This work
pACT3-Pc.tadH-Tt.lac11^{v1}	pACT3 derivative carrying Pc.*tadH* (codon optimized); Tt-*lac11* (codon optimized; Δ1-38 aa)	This work
pEXT22-Ec.mdh^{5Q}	pEXT22 derivative carrying Ec.*mdh*^{5Q} (=Ec.*mdh*$_{I12V:R81A:M85Q:D86S:G179D}$)	This work
pEXT22-Ec.mdh^{5Q}-Hh.araD	pEXT22 derivative carrying Ec.*mdh*^{5Q}, Hh.*araD* (= Hh.*e2k99_19880*)	This work
pEXT22-Ec.mdh^{5Q}-Aa.araD	pEXT22 derivative carrying Ec.*mdh*^{5Q}, Aa.*araD* (= Aa.*acav1654*)	This work
pEXT21-Re.kdgT	pEXT21 derivative carrying Re.kdgT	This work

Supplementary Table 13. Strains for bioproduction efforts used in this study. Sources of employed genes are abbreviated as follows: Ec, *Escherichia coli*; Go, *Gluconobacter oxydans*; Pc, *Paraburkholderia caryophylli*; Tt, *Thermogutta terrifontis*; Hh, *Herbaspirillum huttiense;* Aa, *Acidovorax avenae*; Re, *Ralstonia eutropha*.

Strains	Genotype	Source
**Host strains**		
DH5α	*E. coli fhuA2 Δ(argF-lacZ)U169 phoA glnV44 Φ80 Δ(lacZ)M15 gyrA96 recA1 relA1 endA1 thi-1*	NEB
BL21(DE3)	*hsdR17*	NEB
	E. coli fhuA2 [lon] ompT gal (λ DE3) [dcm] ∆hsdS	
MG1655	*λ DE3 = λ sBamHIo ∆EcoRI-B int::(lacI::PlacUV5::T7 gene1) i21 ∆nin5)*	ATCC 47076
TW63	*E. coli F^{-} λ^{-} ilvG- rfb-50 rph-1*	JMF lab
TW64	MG1655 ∆*yqhD*	JMF lab
TW467	MG1655 ∆*yqhD* ∆*aldA*	This work
TW516	MG1655 ∆*yqhD* ∆*aldA* ∆*lldD* ∆*aspC*	This work
TW1406	MG1655 ∆*yqhD* ∆*aldA* ∆*lldD*	This work
TW1440	MG1655 ∆*yqhD* ∆*aldA* ∆*lldD* ∆*gcl* ∆*glcD*	This work
TW1441	MG1655 ∆*yqhD* ∆*aldA* ∆*lldD* ∆*fsaA* ∆*fsaB*	This work
	MG1655 ∆*yqhD* ∆*aldA* ∆*lldD* ∆*fsaA* ∆*fsaB* ∆*fucO*	
**EG-assimilating strains**		
TW138	TW64 / pEXT20	This work
TW139	TW64 / pEXT20-Ec.fucO	This work
TW140	TW64 / pEXT20-Ec.fucO$^{I6L:L7V}$	This work
TW141	TW64 / pEXT20-Go.adh	This work
TW167	TW63 / pEXT20	This work
TW168	TW63 / pEXT20-Ec.fucO	This work
TW169	TW63 / pEXT20-Ec.fucO$^{I6L:L7V}$	This work
TW170	TW63 / pEXT20-Go.adh	This work
**D-threonate assimilating strains**		
TW270	MG1655 / pEXT22-Re.dtnK-Re.pdxA2-Re.kdgT	This work
TW276	MG1655 / pEXT22-Re.dtnK-Re.pdxA2	This work
**Producer strains**		
TW145	TW64 / pEXT20-Ec.fsaA^{TA}	This work
TW146	TW64 / pEXT20-Ec.fsaA^{TA}-Pc.tadH	This work
TW253	TW64 / pACT3-Ec.fsaA^{TA}-Pc.tadH	This work
TW334	TW64 / pEXT22 / pEXT21	This work
TW335	TW64 / pEXT22-Ec.mdh^{5Q} / pEXT21-Re.kdgT	This work
TW336	TW64 / pEXT22 / pEXT21-Re.kdgT	This work
TW337	TW64 / pEXT22-Ec.mdh^{5Q}-Aa.araD / pEXT21	This work
TW338	TW64 / pEXT22-Ec.mdh^{5Q}-Aa.araD / pEXT21-Re.kdgT	This work
TW339	TW64 / pEXT22-Ec.mdh^{5Q}-Hh.araD / pEXT21-Re.kdgT	This work
TW288	TW64 / pACT3-Ec.fsaA^{TA}-Pc.tadH / pEXT22	This work
TW290	TW64 / pACT3-Ec.fsaA^{TA}-Pc.tadH / pEXT22-Ec.mdh^{5Q}-Hh.araD	This work
TW293	TW64 / pACT3-Ec.fsaA^{TA}-Pc.tadH-Tt.lac11 / pEXT22-Ec.mdh^{5Q}-Hh.araD	This work
TW304	TW64 / pACT3-Ec.fsaA^{TA}-Pc.tadH-Tt.lac11 / pEXT22-Ec.mdh^{5Q}-Hh.araD / pEXT21-Re.kdgT	This work
TW354	TW64 / pACT3-Ec.fsaA^{TA}-Pc.tadH-Tt.lac11^{v1} / pEXT22-Ec.mdh^{5Q}-Hh.araD / pEXT21-Re.kdgT	This work
TW356	TW64 / pACT3-Ec.fsaA^{TA}-Pc.tadH-Tt.lac11^{v1} / pEXT22-Ec.mdh^{5Q}-Hh.araD	This work
TW363	TW64 / pACT3-Go.adh-Ec.fsaA^{TA}-Pc.tadH-Tt.lac11^{v1} / pEXT22-Ec.mdh^{5Q}-Hh.araD / pEXT21-Re.kdgT	This work
TW1354	TW467 / pACT3-Ec.fsaA^{TA}-Pc.tadH-Tt.lac11^{v1} / pEXT22-Ec.mdh^{5Q}-Hh.araD / pEXT21-Re.kdgT	This work
TW1356	TW516 / pACT3-Ec.fsaA^{TA}-Pc.tadH-Tt.lac11^{v1} / pEXT22-Ec.mdh^{5Q}-Hh.araD / pEXT21-Re.kdgT	This work
TW1407	TW1406 / pACT3-Ec.fsaA^{TA}-Pc.tadH-Tt.lac11^{v1} / pEXT22-Ec.mdh^{5Q}-Hh.araD / pEXT21-Re.kdgT	This work
TW1446	TW1440 / pACT3-Ec.fsaA^{TA}-Pc.tadH-Tt.lac11^{v1} / pEXT22-Ec.mdh^{5Q}-Hh.araD / pEXT21-Re.kdgT	This work
TW1447	TW1441 / pACT3-Ec.fsaA^{TA}-Pc.tadH-Tt.lac11^{v1} / pEXT22-Ec.mdh^{5Q}-Hh.araD / pEXT21-Re.kdgT	This work
TW1497	TW337 / pACT3-Pc.tadH	This work
TW1505	TW337 / pACT3-Pc.tadH-Tt.lac11	This work
TW1498	TW337 / pACT3-Pc.tadH-Tt.lac11^{v1}	This work
TW1500	TW339 / pACT3-Pc.tadH	This work
TW1507	TW339 / pACT3-Pc.tadH-Tt.lac11	This work
TW1501	TW339 / pACT3-Pc.tadH-Tt.lac11^{v1}	This work
TW1828	TW516 / pACT3-Go.adh-Ec.fsaA^{TA}-Pc.tadH-Tt.lac11^{v1} / pEXT22-Ec.mdh^{5Q}-Hh.araD / pEXT21-Re.kdgT	This work

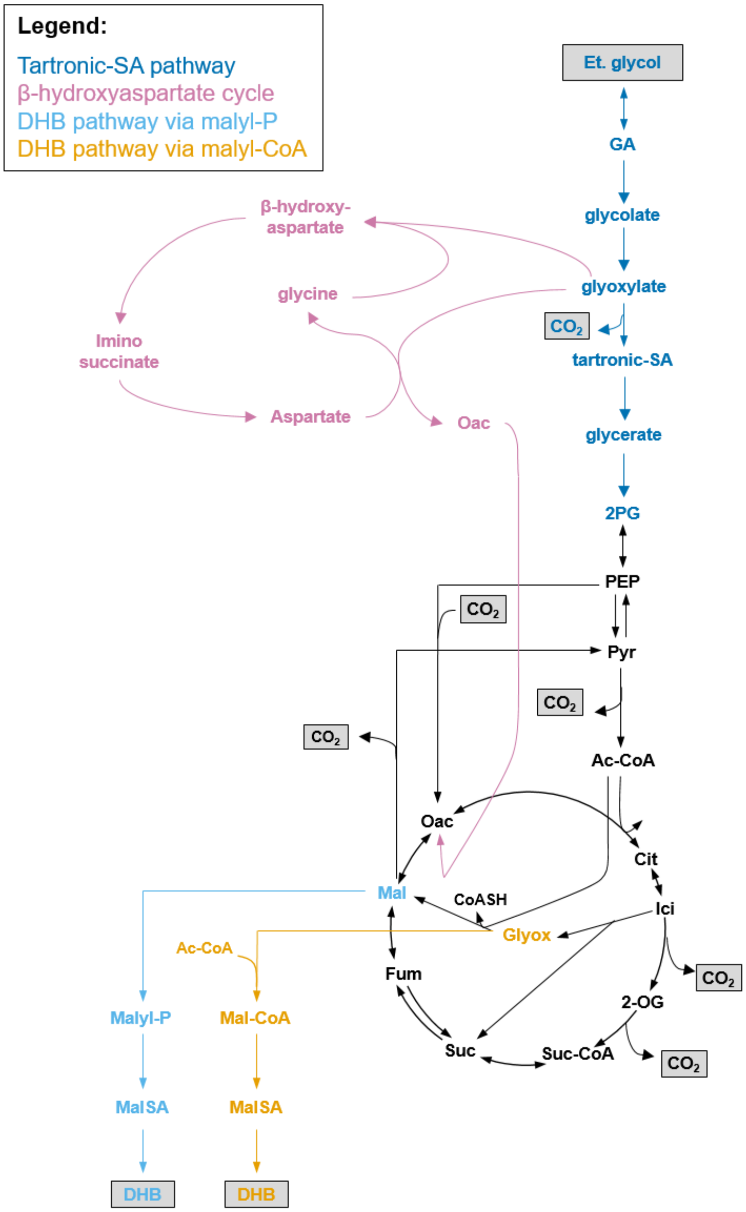

Supplementary Figure 1. Assimilation of ethylene glycol via tartronic-SA natural pathway or β-hydroxyaspartate cycle enables production of intermediate molecules malate and glyoxylate/acetyl-CoA which serve as precursors for DHB synthesis via non-natural malyl-P and/or malyl-CoA pathways, respectively. Theoretical molar DHB yields are identical when operating individually or simultaneously both DHB pathway segments (0.33 mol mol^{-1}). Elementary flux mode analysis[19] was implemented in CellNetAnalyser[20] and used to analyze a previously published stoichiometric model[21] of *E. coli* central carbon metabolism extended with pathways highlighted in figure above. All simulations were limited to aerobic conditions (flux $_{OAA\text{->}Mal}$ = 0). Legend: 2-OG, 2-oxoglutarate; 2PG, glycerate-2P; GA, glycolaldehyde.

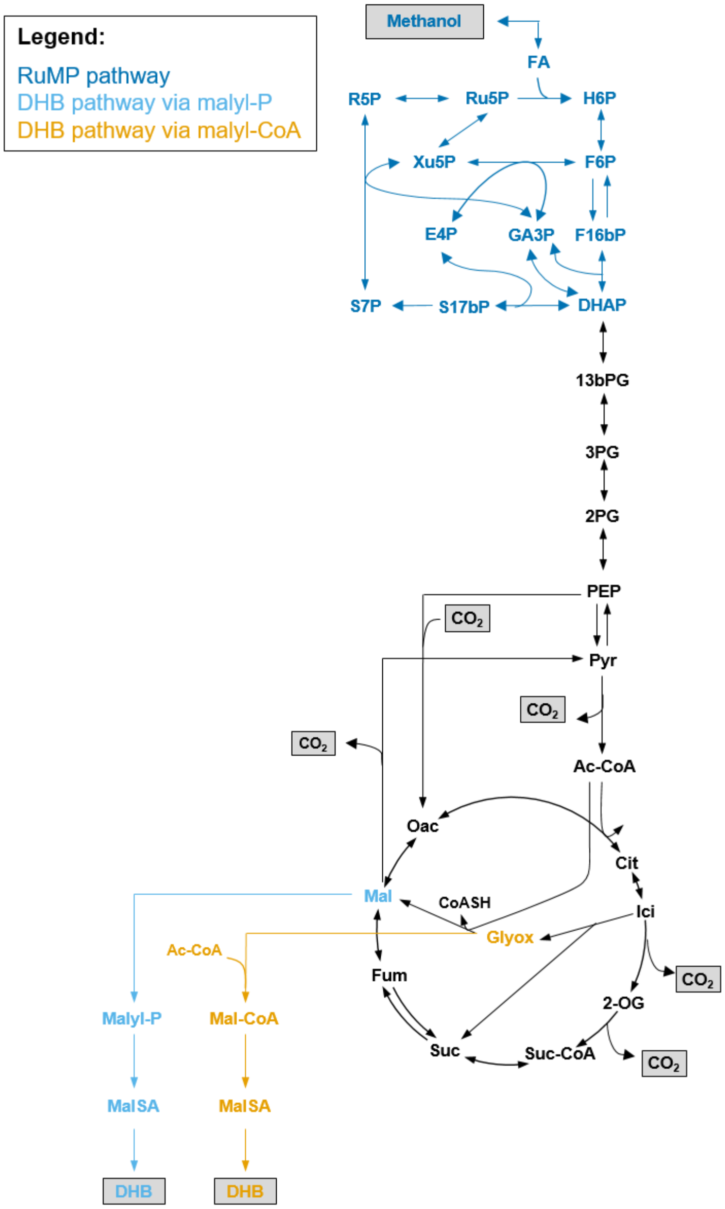

Supplementary Figure 2. Assimilation of methanol via RuMP natural pathway enables production of intermediate molecules malate and glyoxylate/acetyl-CoA which serve as precursors for DHB synthesis via non-natural malyl-P and/or malyl-CoA pathways. Theoretical molar DHB yields when operating individually DHB pathway segments are 0.22 and 0.17 mol mol^{-1} for malyl-P and malyl-CoA pathways, respectively. Simultaneous operation of both pathways does not improve theoretical DHB yield. Elementary flux mode analysis[19] was implemented in CellNetAnalyser[20] and used to analyze a previously published stoichiometric model[21] of *E. coli* central carbon metabolism extended with pathways highlighted in figure above. All simulations were limited to aerobic conditions (flux $_{OAA\text{->}Mal}$ = 0). Legend: 13bPG, glycerate-1,3P; 2-OG, 2-oxoglutarate; 2PG, glycerate-2P; 3PG, glycerate-3P; DHAP, dihydroxyacetone-P; E4P, erythrose-4P; F16bP, fructose-1,6P; F6P, fructose-6P; FA, formaldehyde; GA3P, glyceraldehyde-3P; H6P, 3-hexulose-6P; R5P, ribose-5P; Ru5P, ribulose-5P; S17bP, sedoheptulose-1,7P; S7P, sedoheptulose-7P; Xu5P, xylulose-5P.

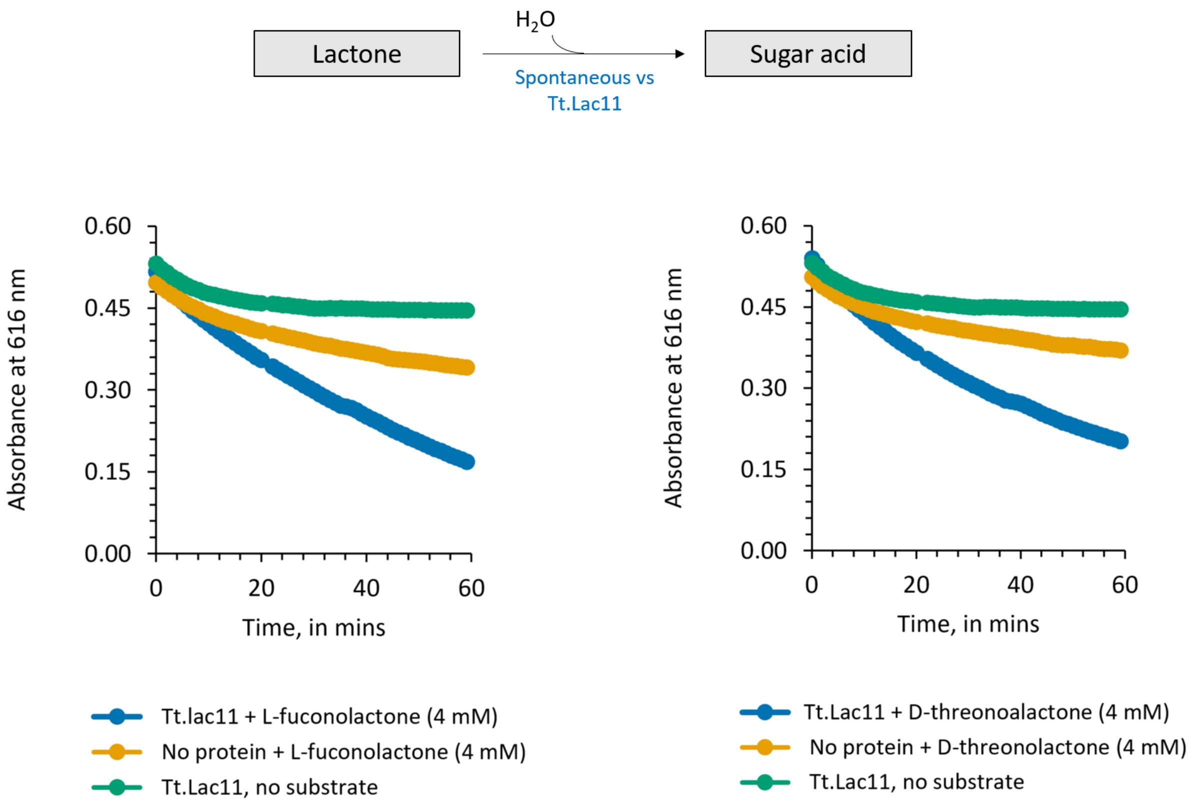

Supplementary Figure 3. Time course for proton release from the carboxylate product in the spontaneous or Tt.Lac11 enzyme-catalysed hydrolysis of lactones (left panel: L-fucono-1,4-lactone; right panel: D-threono-1,4-lactone) as assessed at 616 nm in a colorimetric pH-based assay. The assay was performed at 37 °C in a 0.25 mL reaction mixture containing 2.5 mM Hepes (pH 7.1), 200 mM NaCl, 1% (v/v) DMSO, 0.1 mM bromothymol blue and appropriate amounts of purified enzyme. The reaction was started by adding 4 mM of lactone (blue line). Spontaneous lactone hydrolysis in absence of added enzyme is also shown (red line). A negative control where no substrate is present was also included (green line). Data shown are representative of two independent experiments.

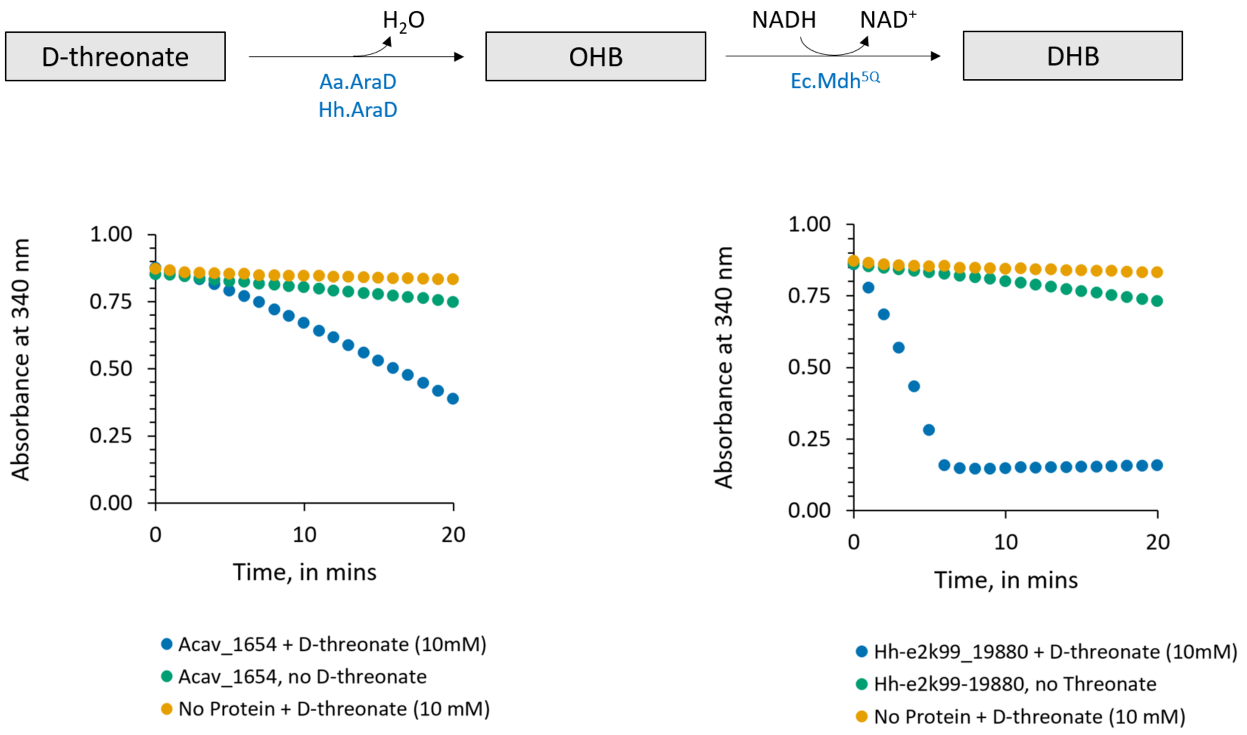

Supplementary Figure 4. Time course for consumption of NADH in the D-threonate dehydratase enzyme assay coupled with OHB reductase Ec-Mdh^{5Q}. The assay was performed at 37 °C in a 0.25 mL reaction mixture containing 60 mM Hepes buffer (pH 7.3), 50 mM KCl, 10 mM $MgCl_2$, 0.25 mM NADH, 100 µg mL^{-1} OHB reductase and appropriate amounts of purified dehydratase enzyme. The reaction was started by adding 10 mM D-threonate (blue circles). Negative controls where no substrate is present (red circles) or no dehydratase was added (green circles) were included. Data shown are representative of two independent experiments.

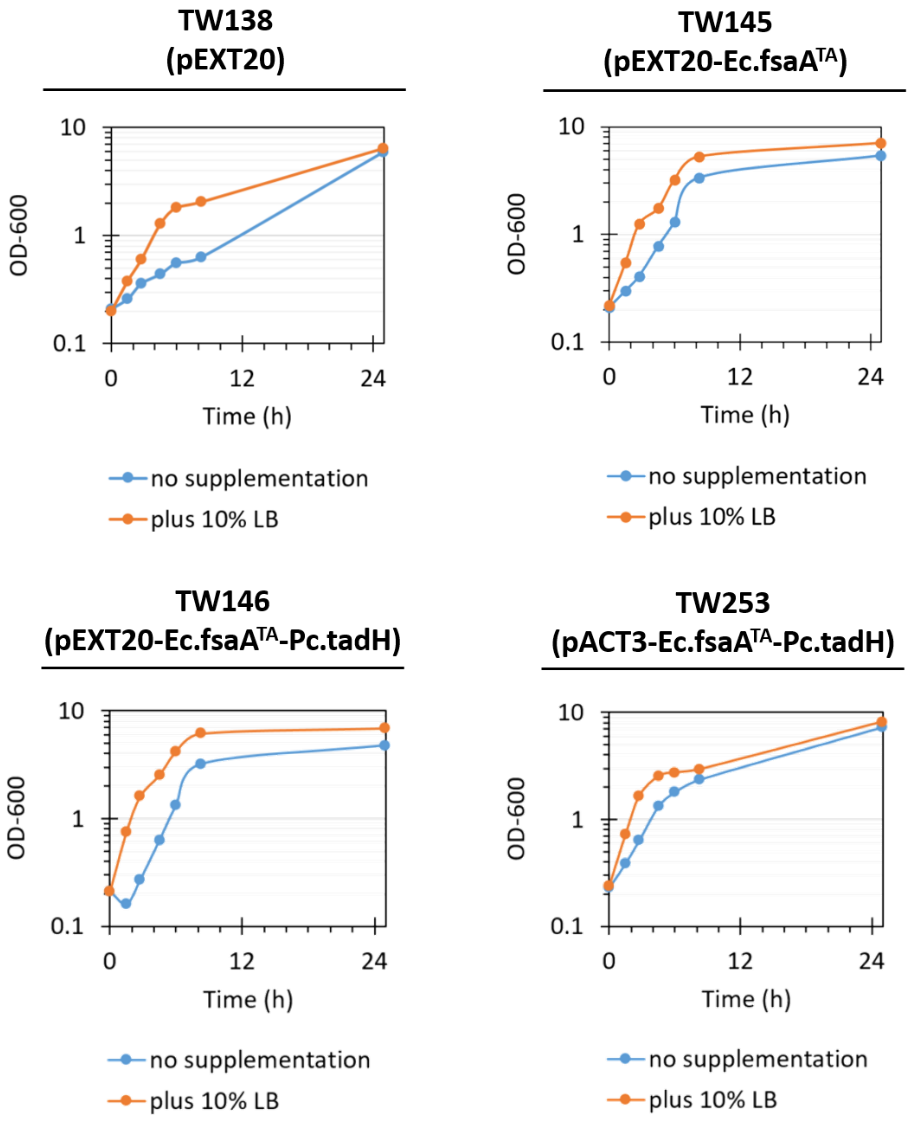

Supplementary Figure 5. Growth curves of D-threose and D-threonate/lactone *E. coli* producer strains cultivated in mineral medium with or without additional supplementation of LB (10 % v/v). Cells were incubated at a starting OD_{600} equal to 0.2. When an OD_{600} ~0.6 was reached, IPTG (0.5 mM) was added. Data shown are representative of two independent experiments.

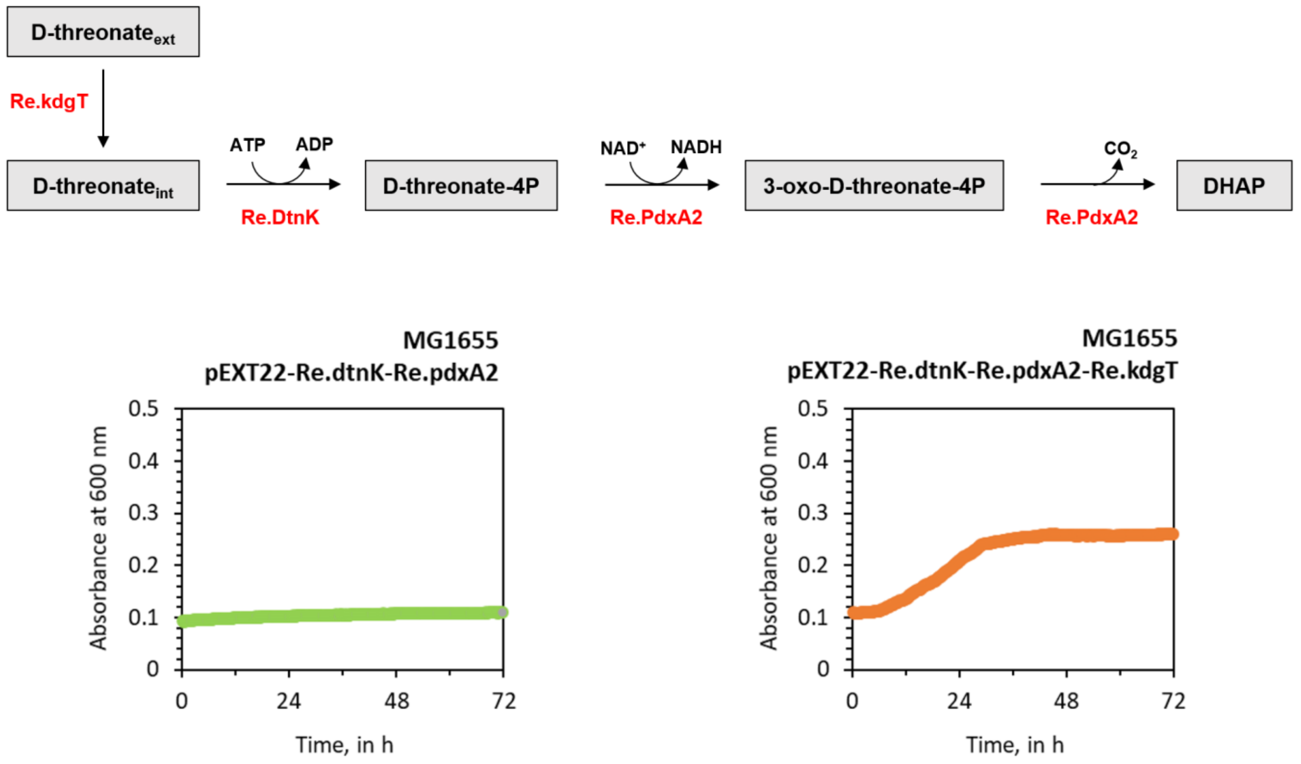

Supplementary Figure 6. Growth of *E. coli* MG1655 harboring a low-copy plasmid pEXT22 expressing threonate kinase (Re.DtnK), threonate-4-P dehydrogenase (Re.PdxA2) and / or threonate importer (Re.KdgT) on 10 mM D-threonate. Cells were pre-cultivated overnight on test tubes containing LB (2 mL) at 37 °C, 220 rpm. Cultures were performed for 72 h on 96-well plates (37 °C, 880 rpm) containing M9 mineral medium containing D-threonate (10 mM) and IPTG (1 mM), to which 50-fold diluted pre-culture (4 µL) was added to each well. Antibiotics were added at standard amounts (kan: 50 µg mL^{-1}). Data shown refer to one single experiment.

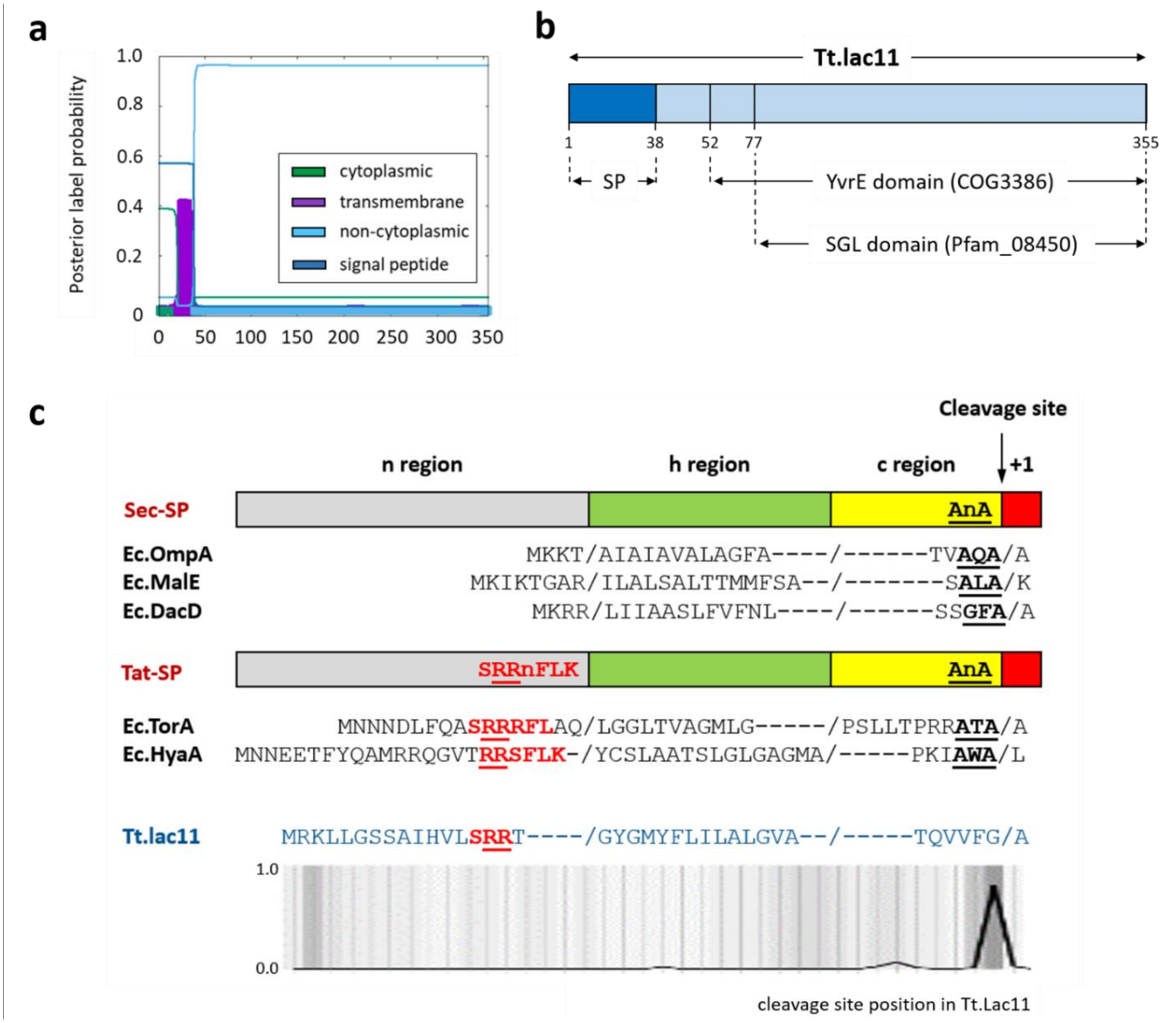

Supplementary Figure 7. Bioinformatic analyses suggest the gluconolactonase from *T. terrifontis* (Tt.Lac11) as a non-cytosolic enzyme. (a) Prediction of protein topology and signal peptide presence using Phobius online server[3] (https://phobius.sbc.su.se/). (b) Schematic representation of Tt.Lac11 amino acid sequence and respective domains (COG, Pfam) identified using BlastP. Signal peptide (SP) is located at N-terminus and was manually assigned based on *in house* analyses herein shown. (c) Sequence alignment between putative signal peptide sequence of Tt.Lac11 and those of periplasmic *E. coli* proteins (OmpA, outer membrane protein A; MalE, maltose ABC transporter periplasmic binding protein; DacD, D-alanyl-D-alanine carboxypeptidase; TorA, trimethylamine N-oxide reductase 1; HyaA, hydrogenase 1 small subunit) targeted to Sec or TAT export pathways (Sec-SP and TAT-SP). A typical signal peptide sequence is distinguished in three regions (n, h, c) whose consensus sequences (as described in work of Palmer *et al.*[22]) are indicated in bold red (wherein n represents a non-conserved amino acid residue). Cleavage site position (37/38; VFG/AE) in Tt.Lac11 was predicted (probability = 0.81; signal peptide likelihood = 0.92) using TargetP-2.0[4] (https://services.healthtech.dtu.dk/service.php?TargetP-2.0).

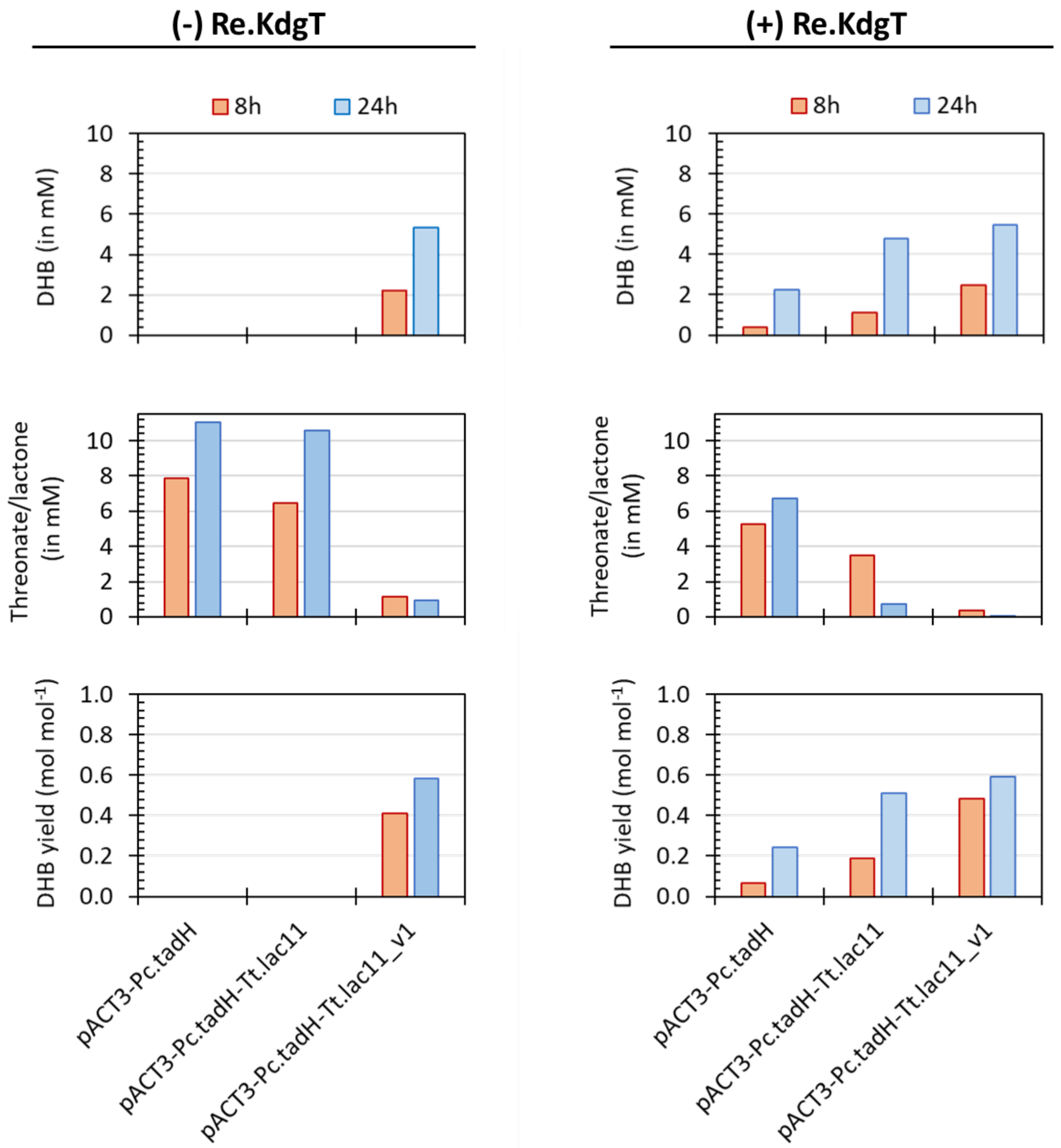

Supplementary Figure 8. Whole-cell bioconversion of D-threose (10 mM) to DHB using modified *E. coli* cells with (left panels) and without (right panels) D-threonate importer Re.KdgT. Strains TW337 (left panel; genotype: MG1655 Δ*yqhD* Δ*aldA* / pEXT22-Ec.mdh^{5Q}-Hh.araD / pEXT21) and TW339 (right panel: MG1655 Δ*yqhD* Δ*aldA* / pEXT22-Ec.mdh^{5Q}-Hh.araD / pEXT21-Re.kdgT) served as genetic backgrounds prior to transformation with medium-copy pACT3 derived plasmid expressing Pc.*tadH* gene. The additional expression of non-cytosolic and cytosolic lactonase variants was evaluated. Cells were incubated in 14 mL test tubes containing mineral medium supplemented with LB (10% v/v) and antibiotics. After 2h of incubation, IPTG (0.5 mM) and D-threose (10 mM) were added. Results are shown for total incubation times of 8 and 24 h, and refer to one single experiment.

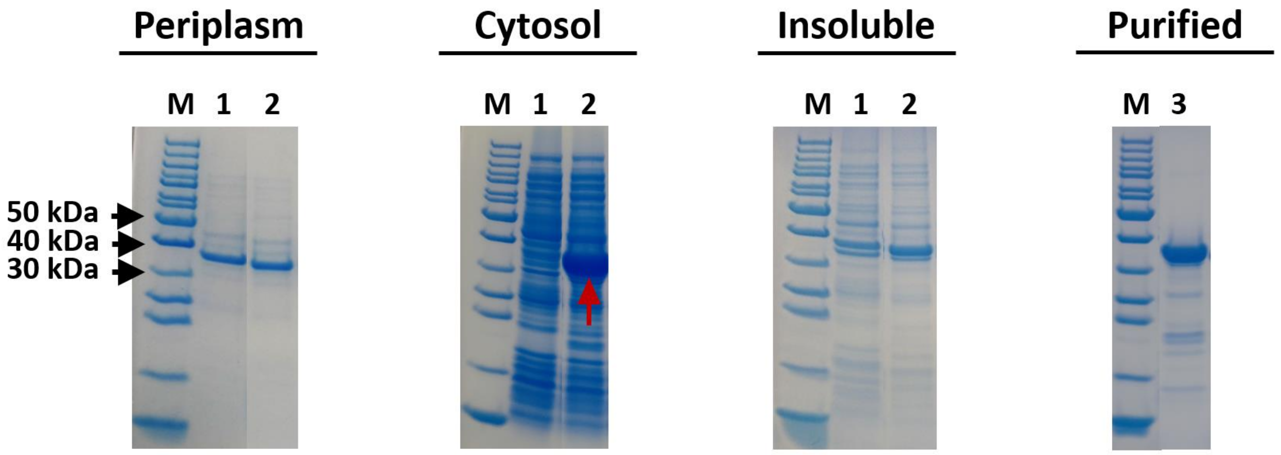

Supplementary Figure 9. SDS-PAGE of subcellular fractions of C-terminus his-tagged cytosolic Tt.Lac11^{v1} lactonase-expressing *E. coli* BL21(DE3) strain and purified enzyme. Legend: M, marker; 1, cells harboring pET28a plasmid; 2, cells harboring pET28a-derived plasmid expressing Tt.Lac11^{v1}; 3, purified Tt.Lac11^{v1}. Source data are provided as a Source Data file.

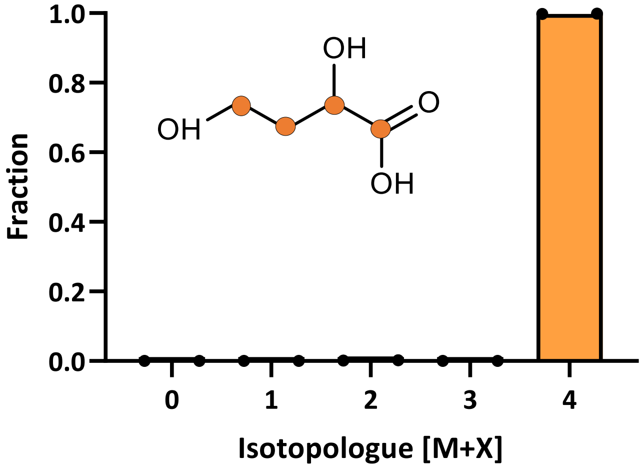

Supplementary Figure 10. Labelling pattern of DHB confirms activity of synthetic pathway. DHB-producing *E. coli* TW354 strain was used in this experiment. Cells were grown in 100 mL shake flasks containing mineral medium supplemented with LB at 10% (v/v). IPTG (0.5 mM) was added when OD_{600} reached ~0.6, and fully labelled ^{13}C-glycolaldehyde (10 mM) was supplied when OD_{600} ~2.0. Total incubation time was 24 h. Relative abundances of extracellularly accumulated DHB isotopologues are illustrated as the mean of two biological replicates. Individual data points are shown as coloured black dots. Source data are provided as a Source Data file.

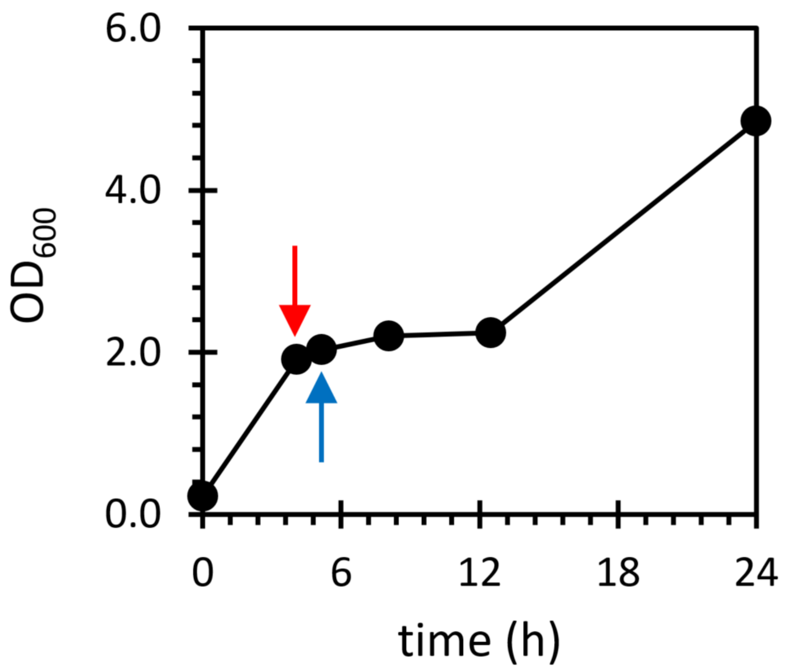

Supplementary Figure 11. Transient growth arrest of strain TW354 after exposure to glycolaldehyde (red arrow). Blue arrow indicates time of cell harvest for intracellular metabolite analysis. Cells were grown in 100 mL shake flasks containing mineral medium supplemented with LB at 10% (v/v). IPTG (0.5 mM) was added when OD_{600} reached ~0.6, and fully labelled ^{13}C-glycolaldehyde (10 mM) was supplied when OD_{600} ~2.0. Results are the mean of two biological replicate experiments. Error bars correspond to the standard deviation of the mean.

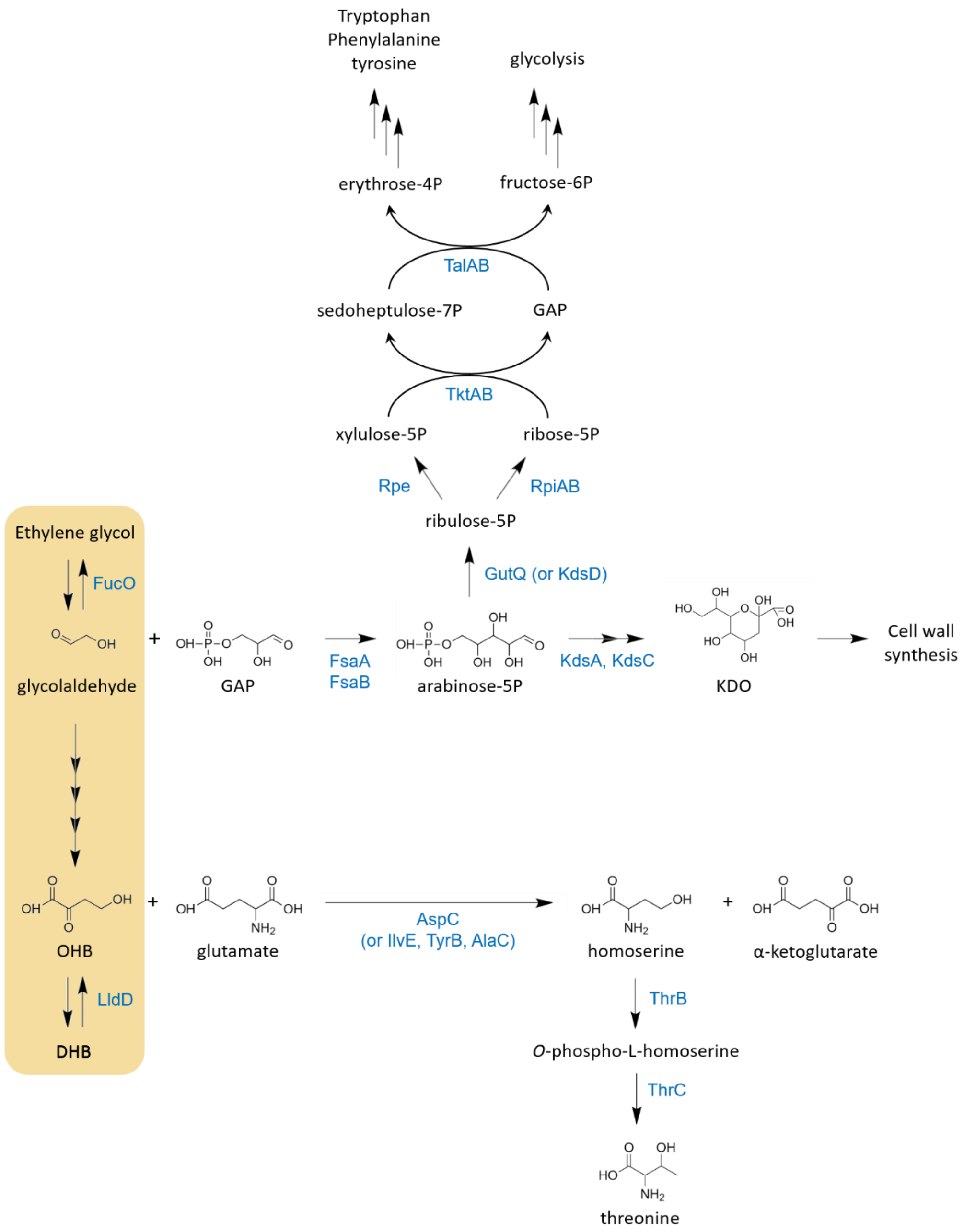

Supplementary Figure 12. Potential carbon sinks of glycolaldehyde-to-DHB pathway in modified *E. coli* cells. Metabolites: DHB, 2,4-dihydroxybutyrate; GAP, glyceraldehyde-3P; KDO, 3-deoxy-α-D-manno-2-octulosonate; OHB, 2-oxo-4-hydroxybutyrate. Enzymes: FsaA, fructose-6P aldolase 1; FsaB, fructose-6P aldolase 2; FucO, L-1,2-propanediol oxidoreductase; GutQ, D-arabinose-5P isomerase; KdsA, KDO-8P synthase; KdsC, KDO-8P phosphatase; KdsD, D-arabinose 5P isomerase; LldD, L-lactate dehydrogenase; Rpe, ribulose-P 3-epimerase; RpiA, ribose-5P isomerase A; RpiB, ribose-5P isomerase B; TalA, transaldolase A; TalB, transaldolase B; ThrB, homoserine kinase; ThrC, threonine synthase; TktA, transketolase 1; TktB, transketolase 2.

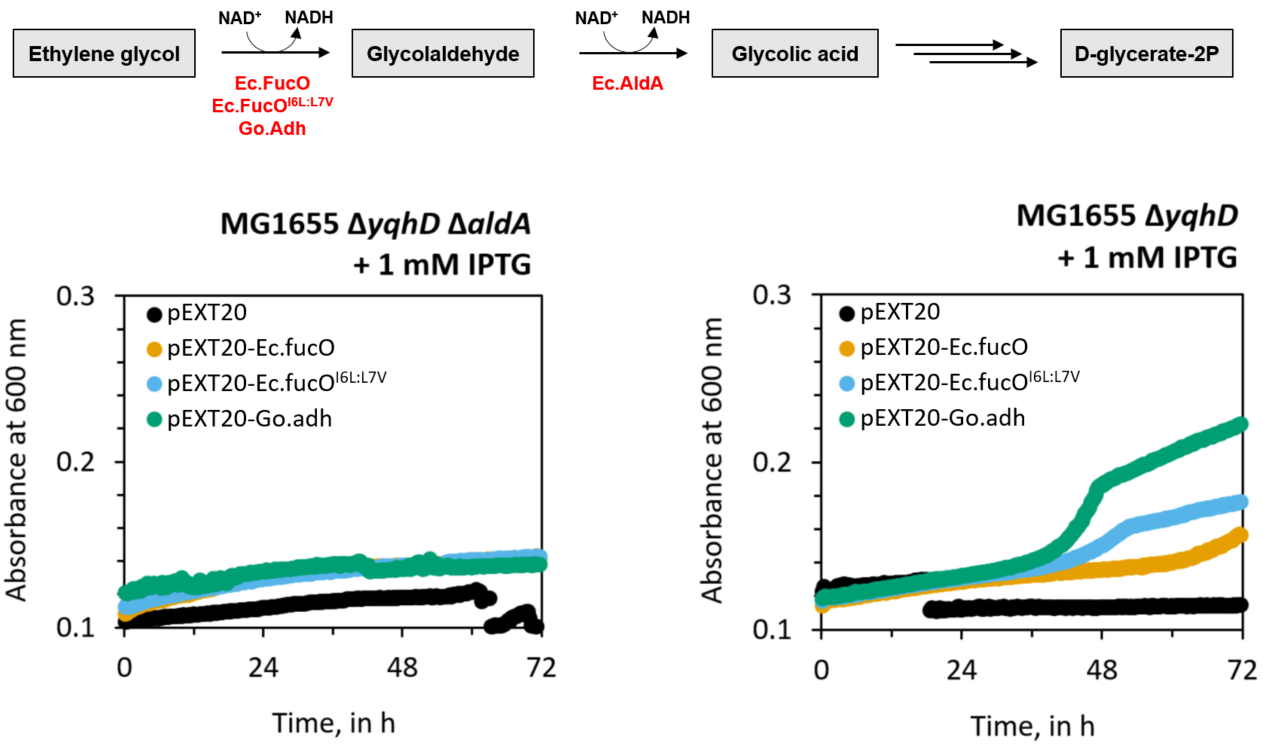

Supplementary Figure 13. Growth on 100 mM ethylene glycol of *E. coli* MG1655 Δ*yqhD* Δ*aldA* (left panel) and Δ*yqhD* (right panel) mutant host strains harboring a high-copy pEXT20 plasmid expressing various ethylene glycol dehydrogenases. Cells were pre-cultivated overnight on test tubes containing LB (2 mL) at 37 °C, 220 rpm. Cultures were performed for 72 h on 96-well plates (37 °C, 880 rpm) containing M9 mineral medium containing ethylene glycol and various amounts of IPTG (mM), to which 50-fold diluted pre-culture (4 µL) was added. Antibiotics were added at standard amounts (amp: 100 µg mL^{-1}). Data shown refer to one single experiment.

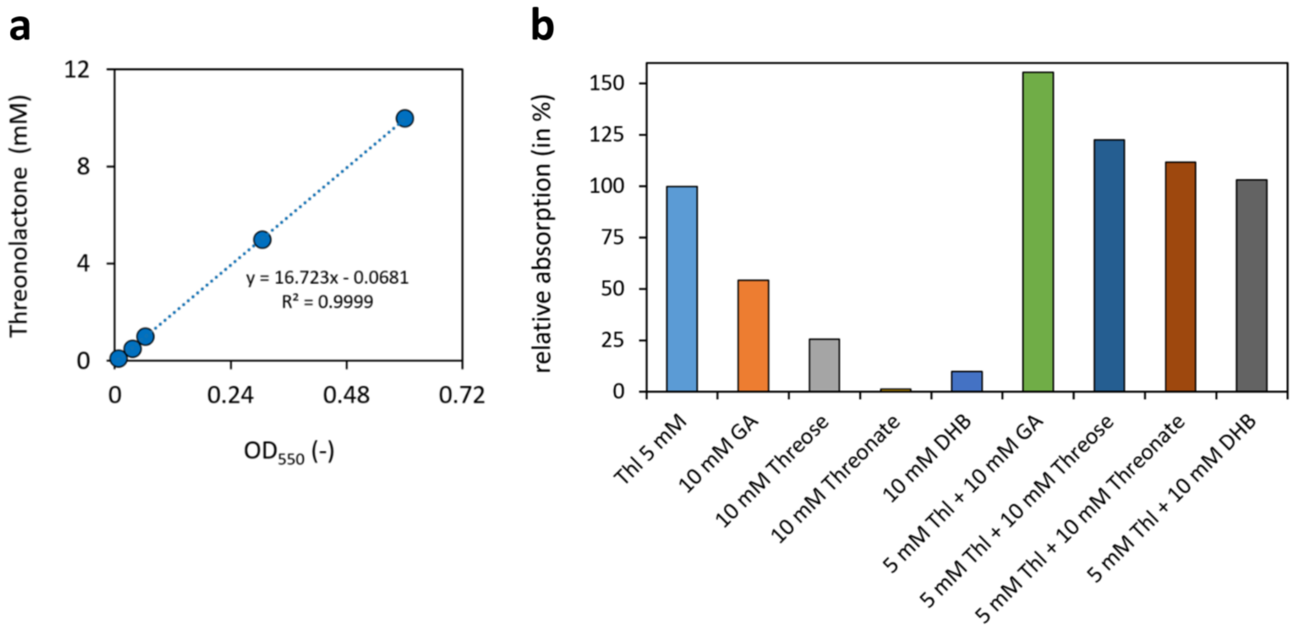

Supplementary Figure 14. Detection of threono-1,4-lactone via colorimetric hydroxamate assay.

(a) Calibration curve using pure standards of threono-1,4-lactone (range: 0.1–10 mM) measured as ferric hydroxamic acid. For a description of the assay, refer to Methods section in main manuscript. Data shown is representative of two independent measurements. (b) Sensitivity test of the assay towards major fermentation co-products of the DHB pathway. Values of OD_{550} were measured when carrying the assay with a pure standard representative of a pathway intermediate alone or together with 5 mM threono-1,4-lactone (Thl). The y-axis displays relative absorption, which corresponds to the percentage of the ratio between measured OD_{550} of ferric hydroxamic acid(s) of tested compound(s) indicated in x-axis and measured OD_{550} values for 5 mM threono-1,4-lactone standard in the form of ferric hydroxamate. Results are shown for one single measurement.

Legend: DHB, 2,4-dihydroxybutyrate; GA, glycolaldehyde; Thl, threono-1,4-lactone.

Supplementary references

1. Jankowski, M. D., Henry, C. S., Broadbelt, L. J. & Hatzimanikatis, V. Group contribution method for thermodynamic analysis of complex metabolic networks. *Biophys. J.* **95**, 1487–1499 (2008).

2. Westlake, A. Thermostable Enzymes Important For Industrial Biotechnology. PhD thesis, https://ore.exeter.ac.uk/repository/handle/10871/37766 (2019).

3. Käll, L., Krogh, A. & Sonnhammer, E. L. L. A combined transmembrane topology and signal peptide prediction method. *J. Mol. Biol.* **338**, 1027–1036 (2004).

4. Armenteros, J. J. A. *et al.* Detecting sequence signals in targeting peptides using deep learning. *Life Sci. Alliance* **2**, e201900429 (2019).

5. Malherbe, G., Humphreys, D. P. & Davé, E. A robust fractionation method for protein subcellular localization studies in *Escherichia coli*. *Biotechniques* **66**, 171–178 (2019).

6. Rollan, C. H. *et al.* Protein expression and extraction of hard-to-produce proteins in the periplasmic space of *Escherichia coli. Protocols.io* https://www.protocols.io/view/protein-expression-and-extraction-of-hard-to-produ-bdr2i58e.html, (2020).

7. Boronat, A. & Aguilar, J. Rhamnose-induced propanediol oxidoreductase in *Escherichia coli*: purification, properties, and comparison with the fucose-induced enzyme. *J. Bacteriol.* **140**, 320–326 (1979).

8. Hobbs, M. E. *et al.* Discovery of an L-fucono-1,5-lactonase from cog3618 of the amidohydrolase superfamily. *Biochemistry* **52**, 239–253 (2013).

9. Tai, Y. S. *et al.* Engineering nonphosphorylative metabolism to generate lignocellulose-derived products. *Nat. Chem. Biol.* **12**, 247–253 (2016).

10. Frazão, C. J. R., Topham, C. M., Malbert, Y., François, J. M. & Walther, T. Rational engineering of a malate dehydrogenase for microbial production of 2,4-dihydroxybutyric acid via homoserine pathway. *Biochem. J.* **475**, 3887–3901 (2018).

11. Dykxhoorn, D. M., St Pierre, R. & Linn, T. A set of compatible tac promoter expression vectors. *Gene* **177**, 133–6 (1996).

12. van Bergen, B., Strasser, R., Cyr, N., Sheppard, J. D. & Jardim, A. α,β-dicarbonyl reduction by *Saccharomyces* D-arabinose dehydrogenase. *Biochim. Biophys. Acta* **1760**, 1636–1645 (2006).

13. Amako, K. *et al.* NAD^+-specific D-arabinose dehydrogenase and its contribution to erythroascorbic acid production in *Saccharomyces cerevisiae*. *FEBS Lett.* **580**, 6428–6434 (2006).

14. Shimizu, T., Takaya, N. & Nakamura, A. An L-glucose catabolic pathway in *Paracoccus* species 43P. *J. Biol. Chem.* **287**, 40448–40456 (2012).

15. Sasajima, K. I. & Sinskey, A. J. Oxidation of L-glucose by a *Pseudomonad*. *Biochim. Biophys. Acta* **571**, 120–126 (1979).

16. Horiuchi, T., Suzuki, T., Hiruma, M. & Saito, N. Purification and characterization of L-fucose (L-

galactose) dehydrogenase from *Pseudomonas* sp. no. 1143. *Agric. Biol. Chem.* **53**, 1493–1501 (1989).

17. Brouns, S. J. J., Turnbull, A. P., Willemen, H. L. D. M., Akerboom, J. & van der Oost, J. Crystal structure and biochemical properties of the D-arabinose dehydrogenase from *Sulfolobus solfataricus*. *J. Mol. Biol.* **371**, 1249–1260 (2007).

18. Watanabe, S., Fukumori, F. & Watanabe, Y. Substrate and metabolic promiscuities of D-altronate dehydratase family proteins involved in non-phosphorylative D-arabinose, sugar acid, L-galactose and L-fucose pathways from bacteria. *Mol. Microbiol.* **112**, 147–165 (2019).

19. Schuster, S., Dandekar, T. & Fell, D. A. Detection of elementary flux modes in biochemical networks: A promising tool for pathway analysis and metabolic engineering. *Trends Biotechnol.* **17**, 53–60 (1999).

20. Klamt, S., Saez-Rodriguez, J. & Gilles, E. D. Structural and functional analysis of cellular networks with CellNetAnalyzer. *BMC Syst. Biol.* **1**, 1–13 (2007).

21. Stelling, J., Klamt, S., Bettenbrock, K., Schuster, S. & Gilles, E. D. Metabolic network structure determines key aspects of functionality and regulation. *Nature* **420**, 190–193 (2002).

22. Palmer, T. & Berks, B. C. The twin-arginine translocation (Tat) protein export pathway. *Nat. Rev. Microbiol. 2012 107* **10**, 483–496 (2012).
