# Supplementary material for: Construction of a synthetic metabolic pathway for biosynthesis of 2,4-dihydroxybutyric acid from ethylene glycol
Source: Nat Commun. 2023 Apr 6;14:1931. doi: 10.1038/s41467-023-37558-x (PMC10079672; doi:10.1038/s41467-023-37558-x)
Supplement: Supplementary file 2 — Description of Additional Supplementary Files [file 41467_2023_37558_MOESM2_ESM.pdf]

Description of Additional Supplementary Files

File Name: Supplementary Data 1
Description: Codon-optimized gene sequences

File Name: Supplementary Data 2
Description: Primers and restriction enzymes used to clone genes coding for candidate genes into pET28a expression vector. Plasmids were constructed via PCR-restriction cloning or homologous recombination employing NEBuilder® HiFi DNA Assembly kit (New England Biolabs).

File Name: Supplementary Data 3
Description: Primers used for construction of plasmids for bioproduction efforts.
